# Frequency‐specific dual‐attention based adversarial network for blood oxygen level‐dependent time series prediction

**DOI:** 10.1002/hbm.70032

**Published:** 2024-09-27

**Authors:** Weihao Zheng, Cong Bao, Renhui Mu, Jun Wang, Tongtong Li, Ziyang Zhao, Zhijun Yao, Bin Hu

**Affiliations:** ^1^ Gansu Provincial Key Laboratory of Wearable Computing, School of Information Science and Engineering Lanzhou University Lanzhou China; ^2^ Second Clinical School Lanzhou University Lanzhou China; ^3^ Department of Magnetic Resonance Lanzhou University Second Hospital Lanzhou China; ^4^ School of Medical Technology Beijing Institute of Technology Beijing China; ^5^ CAS Center for Excellence in Brain Science and Intelligence Technology, Shanghai Institutes for Biological Sciences Chinese Academy of Sciences Shanghai China; ^6^ Joint Research Center for Cognitive Neurosensor Technology of Lanzhou University & Institute of Semiconductors Chinese Academy of Sciences Lanzhou China

**Keywords:** autism spectrum disorder, blood oxygen level‐dependent (BOLD) series prediction, diagnosis, functional magnetic resonance imaging (fMRI), generative adversarial network, major depressive disorder

## Abstract

Functional magnetic resonance imaging (fMRI) is currently one of the most popular technologies for measuring brain activity in both research and clinical contexts. However, clinical constraints often result in short fMRI scan durations, limiting the diagnostic performance for brain disorders. To address this limitation, we developed an end‐to‐end frequency‐specific dual‐attention‐based adversarial network (FDAA‐Net) to extend the time series of existing blood oxygen level‐dependent (BOLD) data, enhancing their diagnostic utility. Our approach leverages the frequency‐dependent nature of fMRI signals using variational mode decomposition (VMD), which adaptively tracks brain activity across different frequency bands. We integrated the generative adversarial network (GAN) with a spatial–temporal attention mechanism to fully capture relationships among spatially distributed brain regions and temporally continuous time windows. We also introduced a novel loss function to estimate the upward and downward trends of each frequency component. We validated FDAA‐Net on the Human Connectome Project (HCP) database by comparing the original and predicted time series of brain regions in the default mode network (DMN), a key network activated during rest. FDAA‐Net effectively overcame linear frequency‐specific challenges and outperformed other popular prediction models. Test–retest reliability experiments demonstrated high consistency between the functional connectivity of predicted outcomes and targets. Furthermore, we examined the clinical applicability of FDAA‐Net using short‐term fMRI data from individuals with autism spectrum disorder (ASD) and major depressive disorder (MDD). The model achieved a maximum predicted sequence length of 40% of the original scan durations. The prolonged time series improved diagnostic performance by 8.0% for ASD and 11.3% for MDD compared with the original sequences. These findings highlight the potential of fMRI time series prediction to enhance diagnostic power of brain disorders in short fMRI scans.

## Graphical abstract

BOLD time series were extracted from preprocessed fMRI data and decomposed into intrinsic mode functions (IMFs) using VMD. Prediction was performed on the IMFs using an adversarial approach and a hybrid loss function. The predicted IMFs were inversely transformed and merged to obtain the final signal sequence of the target region.

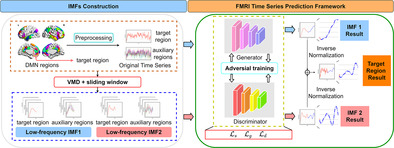

## 1 INTRODUCTION

Resting‐state functional magnetic resonance imaging (rs‐fMRI) measures spontaneous fluctuations of the blood oxygen level‐dependent (BOLD) signal, which has been widely used in mapping functional activation and organization throughout the brain (Liang & Xu, 2022). One major issue for the existing fMRI data is that clinical scans are often limited in duration due to patient constraints, such as those with psychiatric, pregnancy, and elder conditions who cannot tolerate long scans (Schuff et al., 2009), or clinical restrictions, such as budget and staff resources (Chen & Hemmen, 2020). This limitation may pose problems for data analysis including low signal‐to‐noise ratios (Murphy et al., 2007), poor test–retest reliability of functional connectivity (Pannunzi et al., 2017), and limited performance for brain disease diagnosis (Nielsen et al., 2013; Spera et al., 2019). These studies highlight the significance of longer fMRI scans for relevant research. However, prolonging scan time may cause additional burdens on participants and increase the risk of head movement (White et al., 2014). Given the vast amount of short‐scan fMRI data currently available, which is crucial for studying the pathological mechanisms and developing diagnostic methods of brain diseases, exploring ways to extend the time series of existing data without requiring a secondary scan becomes a challenge.

Time series forecasting is essential for analyzing natural and social systems (Liu et al., 2020). Previous studies have adopted various machine learning (ML) algorithms to predict future trends in various fields (Deb et al., 2017; Taieb et al., 2012). Shabbir et al. (Shabbir & Chaturvedi, 2022) used random forest and seasonal autoregressive integrated moving average (ARIMA) to model the historical time series. However, most ML models fail to capture the nonlinearity of real‐world time series. Recently, deep learning (DL) techniques have been successfully applied to address time series forecasting tasks.

Fischera et al. (Fischer & Krauss, 2018) found that long short‐term memory (LSTM) network was superior to the random forest and logistic regression; Wu et al. (2021) designed a transformer model combined with autocorrelation mechanism of the series periodicity. However, most previous studies targeted predictions on a single time sequence, while the brain is a complex network composed of interconnected regions that work synchronously to support various cognitive processes, and thus requires the prediction of multiple simultaneous and interacting time series. Bedel et al. (2023) and Bedel and Çukur (2023) investigated the classification problem of fMRI using transformer and graphical architectures but did not explore time‐series prediction. Sobczak et al. (2021) utilized a recurrent neural network (RNN) to predict the future time‐series of BOLD signals, but could only predict short‐term predictions of 10 s with low accuracy.

**FIGURE 1:**
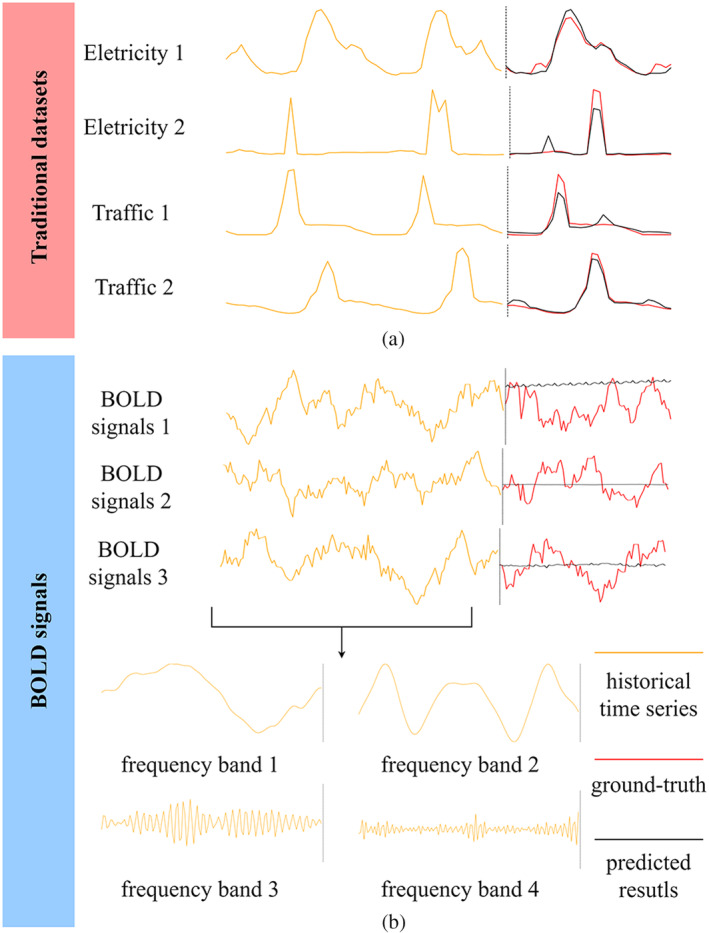
The prediction dilemma of blood oxygen level‐dependent (BOLD) signals.

BOLD signals in different frequency bands represent different information. Low‐frequency signals in the resting state reflect brain activities such as self‐thinking, introspection, and memory, while high‐frequency signals are usually associated with motor control of the brain and execution of tasks (Wu et al., 2008). However, existing studies have only targeted datasets with significant periodicity and a single frequency, such as Traffic and Electricity (Wu et al., 2021; yellow curves in Figure 1a). This leads to the fact that traditional models cannot consider the effect of complex band mixing and struggle to effectively capture the time‐varying patterns when predicting the BOLD signal (black curves in Figure 1b). This leads to models that do not achieve accurate predictions, or even results that present a straight line with almost all features lost, as shown in Figure 1.

Actually, recent studies have focused on the prediction of electrophysiological signals, such as electrocardiogram (ECG; Prakarsha & Sharma, 2022) and electroencephalography (EEG; Gu et al., 2020). However, as far as we know, there are few models designed for the prediction of the BOLD signal in the brain, though it may have great potential to improve the clinical application value of the existing fMRI data on brain disorders. Compared with ECG and EEG signals, fMRI signals have higher spatial resolution, which can precisely locate the activated areas and their cooperative regions in the brain. Therefore, it is necessary to model the BOLD signal from both temporal and spatial perspectives to fully exploit brain interaction patterns. In addition, significant differences between BOLD time series of distinct brain regions have been found, which may be due to the activation degree varying in these regions (Liang & Xu, 2022) and lead to considerable difficulty in building a general architecture for fMRI time series prediction. Since the BOLD signals are nonstationary time series and highly susceptible to fluctuations, it may be useful to learn the overall distribution and fluctuation trend of the BOLD signal of a brain region rather than only capture the continuous changes of time series step‐by‐step. Additionally, the BOLD signal distinguishes itself from other physiological signals by necessitating not only high‐precision time‐step prediction but also accurate prediction of functional connectivity (FC) to fulfill genuine clinical requirements.

In the present study, we proposed a novel DL architecture, named frequency‐specific dual‐attention‐based adversarial network (FDAA‐Net), for predicting the BOLD time series of multiple brain regions within the default mode network (DMN). Given that brain activity occurs across a range of frequencies rather than being confined to a specific frequency, we employed variational mode decomposition (VMD) to adaptively extract the frequency components from the time series and enhance the prediction ability. The generative adversarial network (GAN) architecture was used to trap the overall distribution pattern of a target brain region to fully characterize the global information of the BOLD signal of this region. Because of the high spatial resolution of fMRI and the strong nonlinearity of BOLD signal, we incorporated the dual attention module into the GAN to learn the contribution of different time steps and different brain regions to the prediction task, and assigned distinct weights to brain regions and time points, respectively. We then introduced a trend loss to consider the irregular fluctuation of the BOLD signal. Experimental results showed that the FDAA‐Net achieved the best performance in predicting BOLD time series on the Human Connectome Project (HCP) (Glasser, Smith, et al., 2016) dataset compared with other popular ML and DL methods. We conducted test–retest reliability tests to assess whether the prediction results could effectively replicate FC effects of the original sequences. We also explored the clinical value of our model by conducting classification analysis for autism spectrum disorder (ASD) and major depressive disorder (MDD) based on the prolonged fMRI time series.

## 2 MATERIALS AND METHODS

### 2.1 Data acquisition and preprocessing

The fMRI data we used were downloaded from the Human Connectome Project (HCP). The HCP is a well‐established and widely used neuroimaging database that includes high‐quality multimodal imaging data acquired from thousands of healthy young adults (Glasser, Smith, et al., 2016). The acquisition parameters of the T1‐weighted images are as follows: echo time (TE)/repetition time (TR)  = 2.14 ms / 2400 ms, slice thickness = 0.7 mm, the number of slices = 256, and echo spacing = 7.6 ms. Parameters for the rs‐fMRI are TE /TR = 33.1 ms / 720 ms, slice thickness = 2.0 mm, the number of slices = 72, and echo spacing = 0.58 ms. We excluded 260 subjects due to missing image data, motion artifacts (head movements >1 mm or any *x*, *y*, *z* direction >1°), and the quality control issues mentioned by the HCP wiki (https://wiki.humanconnectome.org/). The remaining 824 healthy subjects (HC) were used for analysis.

We used two additional datasets from the Autism Brain Imaging Data Exchange I (ABIDE I, http://fcon_1000.projects.nitrc.org/indi/abide/abide_I.html) and the REST‐meta‐MDD dataset (http://rfmri.org/REST-meta-MDD; Yan et al., 2019) to evaluate the clinical utility of our model in brain disorder diagnosis. We selected the University of Michigan site of ABIDE I (ABIDE I‐UM) with 54 subjects (20 patients with ASD and 34 HC) and the Institute of Mental Health site of REST‐meta‐MDD (REST‐meta‐MDD‐IMH) with 44 subjects (25 patients with MDD and 19 HC). The subjects with excessive head motion and IQ < 70 (for the ABIDE I‐UM) or HAMD total score <7 (for the REST‐meta‐MDD‐IMH) were excluded. As for the ABIDE I‐UM, the parameters for the fMRI are as follows: TR = 2000 ms, TE = 30 ms, field of view (FOV) = 22 mm, thickness = 3 mm, number of slices = 40; T1‐weighted MRI images were acquired with the following parameters: TR = 250 ms, TE = 5.7 ms, flip angle = 90°, and thickness = 3 mm. The fMRI parameters of the REST‐meta‐MDD‐IMH are TR/TE = 2000/30 ms, thickness = 4 mm, number of slices = 36; and T1‐weighted MRI were acquired by using the parameters: TR/TE = 7.5/3.7 ms, flip angle = 8°, and thickness = 1 mm. The demographic information of the enrolled participants is given in Table 1.

**TABLE 1:** Demographic characteristics of the enrolled subjects.

Dataset	HCP	ABIDE I‐UM		REST‐meta‐MDD‐IMH	
		ASD	HC	MDD	HC
Gender (M/F)	363/461	16/4	24/10	12/13	11/8
Age	28.65 ± 3.79	13.73 ± 2.38	14.29 ± 3.10	35.20 ± 9.88	24.47 ± 7.04
Clinical assessment	—	44.10 ± 8.69 (ADOS)	—	23.04 ± 5.30 (HAMD)	—

Abbreviations: ADOS, autism diagnostic interview‐revised score; ASD, autism spectrum disorder; HAMD, Hamilton depression rating scale score; HCP, Human Connectome Project; MDD, major depressive disorder; M/F, male/female.

The HCP minimal preprocessing pipeline was used to preprocess rs‐fMRI data (Glasser et al., 2013), followed by the application of the independent component analysis (ICA) based Xnoiseifier (FIX) to remove artifacts from the processed data (Salimi‐Khorshidi et al., 2014). The Data Processing and Analysis of Brain Imaging (DPABI) toolbox (Yan et al., 2016) was used for the preprocessing of images from the other two clinical datasets. The preprocessing steps included slice timing, head motion correction, normalization, spatial smoothing, filtering (0.01–0.1 Hz), the regression of the global signal regression, and average signals from ventricles and white matter. Subjects with head movements >1 mm or any *x*, *y*, *z* direction >1° were excluded. We also conducted the same regression and smooth procedure on HCP data. We used the Human Connectome Project‐MultiModal Parcellation (HCP‐MMP 1.0) atlas (Glasser, Coalson, et al., 2016) to segment the cortex into 360 brain regions for HCP dataset. The automated anatomical labeling (AAL) atlas (Rolls et al., 2015) was also used to evaluate the robustness of the proposed model under different parcellations. Since the DMN is the most robust, activated, and interconnected system in the resting state (Hacker et al., 2013), we selected 16 regions from the HCP‐MMP atlas or 10 regions from the AAL atlas to construct the DMN, and then extracted the average time sequence of each region as the prediction target.

### 2.2 Overview of the time series prediction framework

**FIGURE 2:**
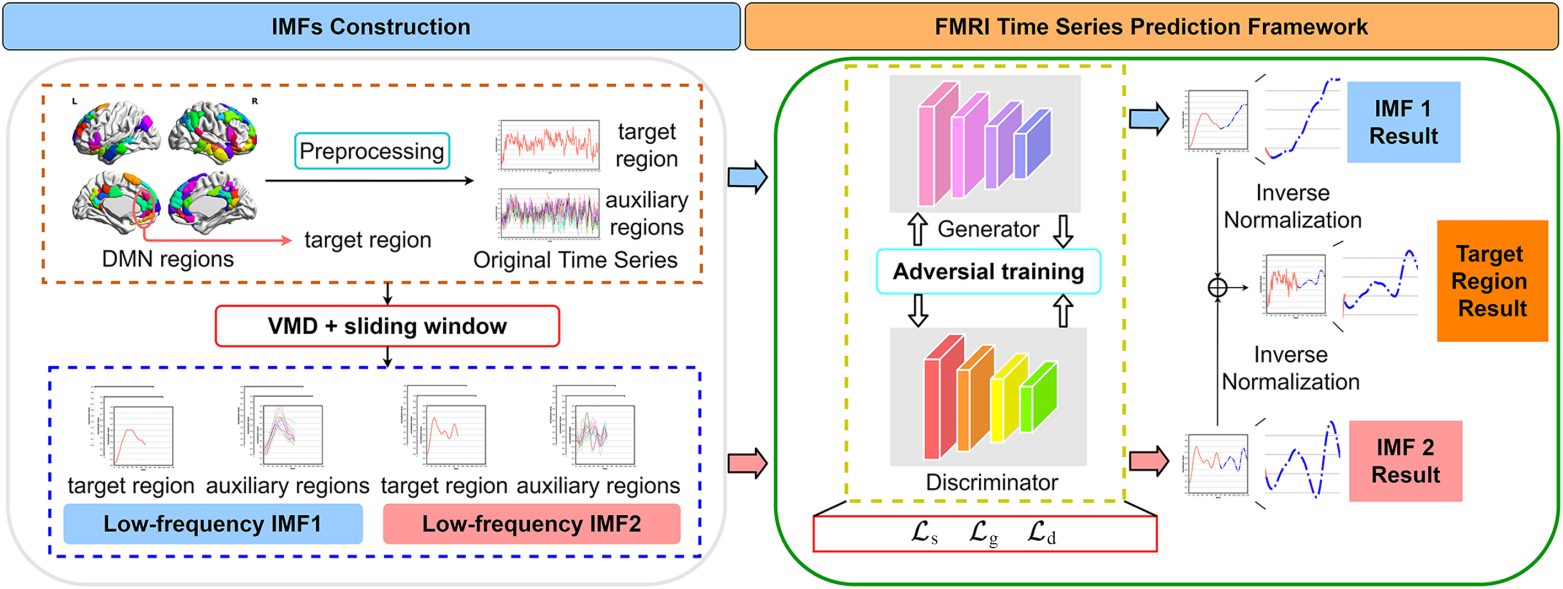
The flowchart of blood oxygen level‐dependent (BOLD) time series prediction. BOLD time series were extracted from preprocessed functional magnetic resonance imaging (fMRI) data and decomposed into intrinsic mode functions (IMFs) using variational mode decomposition (VMD). Prediction was performed on the IMFs using an adversarial approach and a hybrid loss function. The predicted IMFs were inversely transformed and merged to obtain the final signal sequence of the target region. DMN, default mode network.

Figure 2 illustrates the flowchart of the proposed DL model. The functional time series were extracted from the preprocessed fMRI data. Then we applied VMD to decompose the original signal into a series of components in different frequency bands, which are called “intrinsic mode functions” (IMFs). Because the functional connectome is stable and highly reproducible in the low‐frequency band (e.g., 0.01–0.05 Hz) across different acquisition sites (Wang et al., 2023), we chose the IMFs in the range of 0.01–0.05 Hz for further analysis. More details about the reason could be seen in discussion. The prediction was performed on each of the chosen IMF components, respectively. The DMN regions were alternately selected as the target region for time series prediction. The IMF series of the target region and the other DMN regions (referred to as auxiliary regions) were simultaneously fed into the model. The time series prediction model adopted an adversarial approach to address the limitation of traditional models that did not consider the global features of a time series, such as the distribution and shape of input data. To enhance the sensitivity of the model to the trend changes of the time series, we incorporated a trend loss that considered the incremental or decremental nature of the adjacent time points into the loss function. The final loss function was the combination of adversarial and supervised loss functions. The predicted IMFs were then inversely transformed into signals and merged to form the final predicted signal sequence.

### 2.3 Problem definition

The time series was segmented using a sliding window method, with the window length L=D+H, where D is the length of historical time points and H is the forecasting horizon. Each time point implies one time step, and N is the last time step of the historical time sequence. $\left[Y_{N-D+1},Y_{N-D+2},\cdots ,Y_{N}\right]$ denotes an input historical time series and $\left[Y_{N+1},Y_{N+2},\cdots ,Y_{N+H}\right]$ is an output that denotes the predicted values of H steps. We defined $Y={\left(y^{1},y^{2},\cdots ,y^{m}\right)}^{T}$, where m is the number of brain regions, y is the time sequence of one brain region, and T is the number of time points. Suppose the dataset contains M subjects, $\hat{f}$ indicates the estimated model between past and future observations, $Y_{t}$ and ${\hat{Y}}_{t}$ refers to the actual and forecasted value of the time series at the *t*th step, respectively. The prediction model for the *q*th brain region of the *p*th subject is defined as follows:

(1) $$\left[{\hat{y}}_{N+1}^{q},{\hat{y}}_{N+2}^{q},\cdots ,{\hat{y}}_{N+H}^{q}\right]={\hat{f}}_{p}^{q}\left(Y_{N-D+1},Y_{N-D+2},\cdots ,Y_{N}\right).$$

### 2.4 Variational mode decomposition

The VMD is a nonrecursive, data‐driven method that adaptively decomposes the signal into a series of IMF components (Dragomiretskiy & Zosso, 2013). Previous studies have demonstrated its remarkable ability to improve the performance of prediction been widely used for decomposing non‐stationary time series prediction tasks due to (Lahmiri, 2016; Rayi et al., 2022). Previous studies have demonstrated distinct BOLD signal oscillation in different frequency bands (Yuen et al., 2019). Therefore, we used VMD to simplify the prediction model of the BOLD signal.

Denotes the original signal as $x\left(t\right)$, which can be decomposed into several IMFs ($u_{k}$) via the VMD, with $u_{k}$ corresponding to a central frequency $\omega_{k}$. The VMD decomposition process includes three steps: (1) for each IMF $u_{k}$, the Hilbert transform is performed to estimate the unilateral frequency spectrum; (2) the frequency bands of all modes are shifted to the baseband and the center of mass of the positive part of the power spectrum is considered as central frequency $\omega_{k}$; (3) the bandwidth of each mode is estimated via the $H^{1}$ Gaussian smoothness. The variational constraint of the problem is as follows:

(2) $$\left\{\begin{array}{c} \min_{\left\{u_{k}\right\},\left\{\omega_{k}\right\}}\left\{\sum_{k=1}^{K}{\left\|\partial_{t}\left[\left(\delta \left(t\right)+\frac{j}{\mathrm{\pi t}}\right)*u_{k}\left(t\right)e^{-j\omega_{k}t}\right]\right\|}_{2}^{2}\right\}\\ s.t.\sum_{k=1}^{K}u_{k}\left(t\right)=x\left(t\right) \end{array}\right..$$

where $\delta \left(t\right)$ is Dirac's delta function, 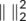 is the Euclidean norm squared, * is a convolution operator, K is the number of IMFs.

To address the variational constraint problem mentioned in equation 2, Dragomiretskiy and Zosso (2013) proposed a new constraint function by introducing Lagrange multipliers λ.

(3) $$\begin{array}{l} ℒ\left(\left\{u_{k}\right\},\left\{\omega_{k}\right\},\lambda \right)≔\alpha \sum_{k}{\left\|\frac{d}{\mathrm{dt}}\left[\left(\delta \left(t\right)+\frac{j}{\mathrm{\pi t}}\right)*u_{k}\left(t\right)\right]e^{-j\omega_{k}t}\right\|}_{2}^{2}\\ \;+{\left\|x\left(t\right)-\sum_{k}u_{k}\right\|}_{2}^{2}+\left\langle \lambda \left(t\right),x\left(t\right)-\sum_{k}u_{k}\left(t\right)\right\rangle . \end{array}$$

where α is a quadratic penalty parameter that adjusts the weight of the first term to reduce the signal reconstruction error in the noisy environment.

Next, the saddle point of the Lagrangian expression was considered as the solution to the constrained problem, which can be solved iteratively via the alternate direction method of multipliers. Parameters $u_{k}$ and $\omega_{k}$ can be updated by the following formulas:

(4) $$u_{k}^{n+1}\left(\omega \right)=\frac{x\left(\omega \right)-\sum_{i\neq k}u_{i}\left(\omega \right)+\frac{\lambda \left(\omega \right)}{2}}{1+2\alpha {\left(\omega -\omega_{k}\right)}^{2}},$$

(5) $$\omega_{k}^{n+1}=\frac{\int_{0}^{\infty }\omega {\left|u_{k}^{n+1}\left(\omega \right)\right|}^{2}\mathrm{d\omega}}{\int_{0}^{\infty }{\left|u_{k}^{n+1}\left(\omega \right)\right|}^{2}\mathrm{d\omega}}.$$

**FIGURE 3:**
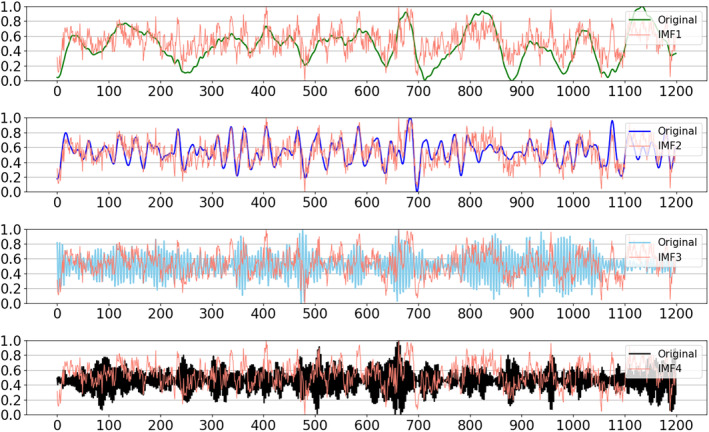
The intrinsic mode functions (IMFs) for one subject in Human Connectome Project (HCP) dataset. For each subgraph, the red curve denotes the original time series, and the other curve represents the signals of IMFs.

We set K=4 and the obtained four IMFs of one HCP subject are visualized in Figure 3. The experimental details are given in Table S1 and Figure S1. The IMF 1 and IMF 2, which had the frequency below 0.05 Hz, were selected as the primary components for the subsequent experiments.

### 2.5 The FDAA‐net model

**FIGURE 4:**
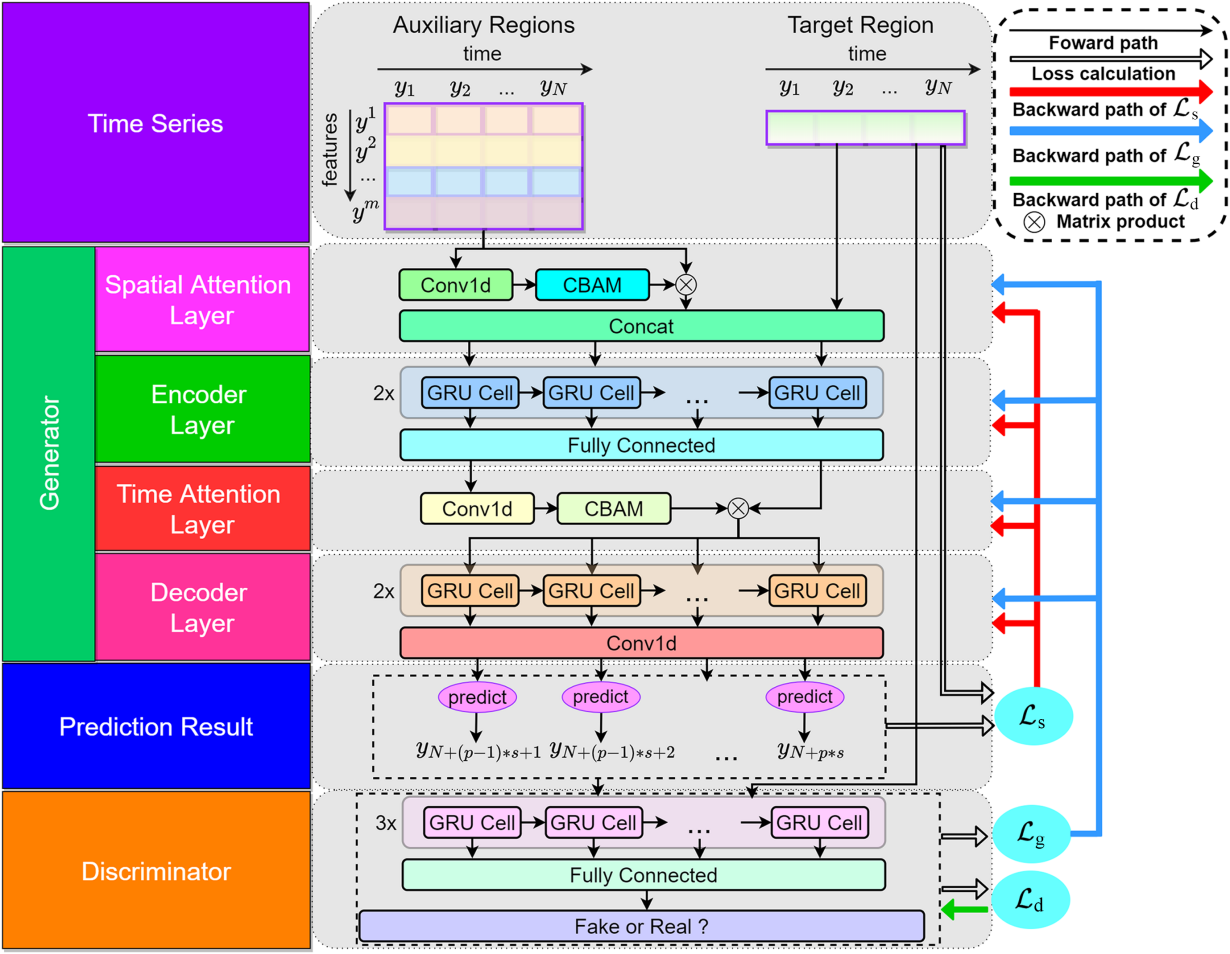
The architecture of the forecasting model. The architecture employed the adversarial paradigm which contained the discriminator (in orange box) and the generator (in green box). The generator was combined with dual attention module which included temporal and spatial attention layers (in red and pink boxes). CBAM, convolutional block attention module; GRU, gated recurrent unit.

Time series of medical data are characterized by high dimensionality and stochasticity nature (Chu et al., 2020), making the prediction task quite challenging. To address this issue, we adopted the adversarial strategy in our prediction model that consisted of the generator (G) and discriminator (D), and these two networks were trained in an alternating manner (see Figure 4). During the training process, the generator generated fake sequences that were as close to the real sequence as possible, and the discriminator was trained to enhance the distinguishing ability of the model between real and predicted sequences. By engaging in a mutually adversarial game, our model extracted global features of the historical time series that capture important distribution and shape information, thereby mitigating potential bias in the prediction task. Therefore, the adversarial strategy enables the model to learn intricate patterns and characteristics of BOLD signals that failed to be captured by traditional forecasting models.

#### 2.5.1 Generator

The generator framework consists of two neural networks, including the dual attention module and the encoder–decoder network.

**FIGURE 5:**
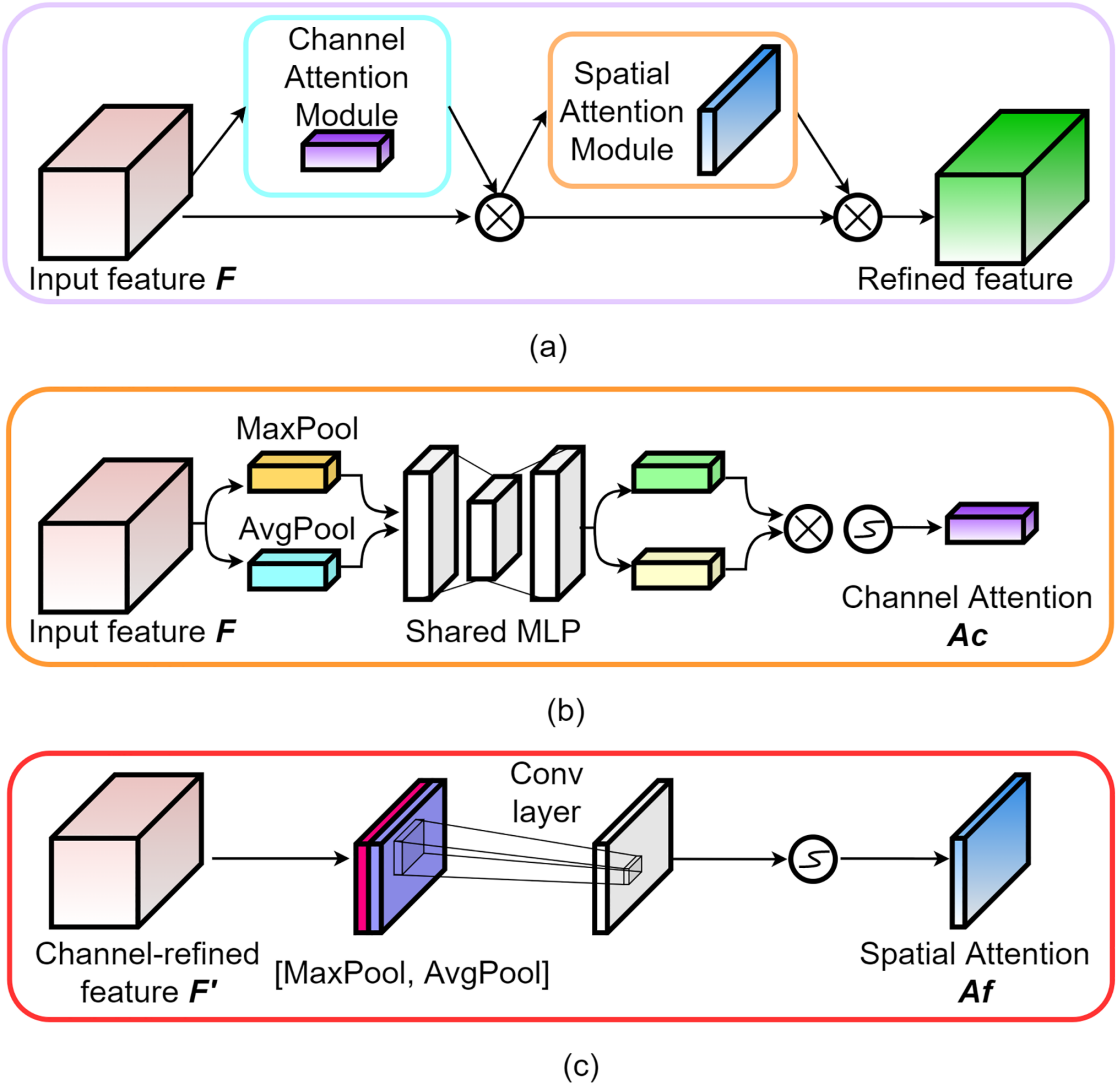
The architecture of the (a–c) convolutional block attention module (CBAM) module. CBAM incorporated both channel and spatial attention modules. MLP, multilayer perception.

- *Dual attention module*: Since fMRI signals exhibit temporal continuity and relevance, and are spatially coherent among the distributed brain regions (e.g., functional connectivity; Jin et al., 2020), the dual attention module was designed to capture the underlying relationship in both spatial and temporal dimensions of the historical time series. Specifically, the dual attention module incorporated temporal and spatial attention layers. The temporal attention layer extracted attention features of different moments in the historical time series, enabling the model to weight the input time windows in the temporal dimension. This facilitated the identification of relevant patterns and trends of time series, while discarding noise and irrelevant information. The spatial attention layer extracted attention features of brain regions that represent the contribution of these regions to the prediction of the BOLD signal of the target region, and the contribution weights can be used to assess the consistency of the results with existing medical physiological knowledge. The temporal and spatial attention layers were composed of an one‐dimensional convolutional network and a convolutional block attention module (CBAM; Woo et al., 2018). The CBAM is a popular attention mechanism that contains the channel attention module (CAM) and spatial attention module (SAM). CAM generates the channel attention $A_{c}$ by feeding the input feature F into both average and max pooling along the channel axis and then submitting the results to the multilayer perception (MLP). The $A_{c}$ element‐wise multiplies the input feature F to generate an intermediate map $F^{\prime }$, which is fed into the SAM to obtain the feature (temporal or spatial) attention value $A_{f}$ through maximum pooling, average pooling, and concatenating the processing results to the convolution layers, respectively. The one‐dimensional convolution kernels were sized in 1×m and N×1, respectively, and we adopted rectified linear unit (ReLU) as the activation function. The framework of CBAM is shown in Figure 5.
- *Encoder–decoder network*: The results from the spatial attention layer were mapped to a high‐dimensional latent space via the encoding layer that adequately populated the feature information learned by the model. The decoding layer generated the predicted time series using the gated recurrent unit (GRU), due to its efficacy in capturing semantic associations among long‐time sequences and mitigating the issues of gradient disappearance or explosion phenomenon (Gao & Glowacka, 2016). Moreover, the GRU requires fewer parameters, which could speed up the training process meanwhile reduce the risk of overfitting. The encoder and decoder layers combined a two‐layer GRU module with a fully‐connected layer and a one‐dimensional convolutional network (1×m kernel size).

included temporal and spatial attention layers (in red and pink boxes).

#### 2.5.2 Discriminator

A typical GRU architecture was constructed for the discriminator network, including the GRU module and the fully‐connected layer. The window‐level *di*scriminator was used to judge the authenticity of each input window. The number of the GRU layers was three and the activation function was ReLU. In the last fully‐connected layer, a sigmoid activation function was adopted to ensure the output range between 0 and 1.

#### 2.5.3 Input and output

The predictions were made at both the subject and IMF levels, meaning that each prediction was generated between sequences belonging to the same IMF within the same subject. Each prediction takes input sequences from all regions of the DMN and designates specific brain regions as targets, while utilizing the remaining brain regions as auxiliary inputs. For instance, consider the left medial prefrontal lobe of the first subject within the DMN. In this scenario, the historical time series of the left medial prefrontal lobe was used as the target sequence, while the historical time series of other brain regions within the DMN acted as auxiliary sequences. These sequences were collectively fed into the model as inputs. Subsequently, the model performed complex computations to generate sequences representing future time periods that were unknown to the model during training. These predicted sequences were then compared against the actual ground‐truth sequences to calculate the loss function and optimize the model during training. Upon completion of model training and testing, the trained prediction model was applied. Specifically, the model was fed with the last time period of the original sequence as input. The output of the model was a sequence that did not exist in the real world, thus enabling the extension of the sequence.

### 2.6 Loss function

The optimization process of traditional prediction models is to minimize a specific loss function such as mean square error (MSE), which may not be sufficient to capture the intricate patterns and dependencies of the fMRI time series. Therefore, we proposed a hybrid loss function involving two components—adversarial loss ${ℒ}_{\mathrm{adv}}$ and supervised loss ${ℒ}_{s}$—to facilitate the characterization of the sophisticated data and thus improve the prediction performance.

#### 2.6.1 The adversarial loss

To implement adversarial training for the generators and discriminators, we split the adversarial loss ${ℒ}_{\mathrm{adv}}$ into a generator loss function ${ℒ}_{g}$ and a discriminator loss function ${ℒ}_{d}$.

The loss function ${ℒ}_{g}$ of the generator was designed to mislead the discriminator. Due to the fact that the sigmoid cross‐entropy loss function is specifically designed for binary classification problems, we established ${ℒ}_{g}$ using the following sigmoid cross‐entropy loss function (Yoon et al., 2019).

(6) $${ℒ}_{\mathrm{sce}}\left(A,B\right)=-\sum_{i}B_{i}\log\left(\text{sigmoid}\left(A_{i}\right)\right)+\left(1-B_{i}\right)\log\left(1-\text{sigmoid}\left(A_{i}\right)\right),$$

(7) $${ℒ}_{g}\left(\hat{y}\right)=L_{\mathrm{sce}}\left(D\left(\hat{y}\right),1\right).$$

The discriminator was trained by using the loss function ${ℒ}_{d}$, which minimizes the probability of making incorrect decisions by the discriminator. The discriminator compared the difference between the real sequence and the generated sequence to calculate a gradient which was used as feedback to modulate the generator in order to generate more accurate predictions. The ${ℒ}_{d}$ is defined as:

(8) $${ℒ}_{d}\left(y,\hat{y}\right)=={ℒ}_{\mathrm{sce}}\left(D\left(\hat{y}\right),0\right)+{ℒ}_{\mathrm{sce}}\left(D\left(y\right),1\right).$$

#### 2.6.2 The supervised loss

However, only optimizing the adversarial loss may not be enough to achieve an accurate prediction. Due to the potential for the generated data to mislead the discriminator while still remaining substantially different from the real data. To overcome this problem, a hybrid supervised loss function ${ℒ}_{s}$ was proposed, which consists of an absolute error loss ${ℒ}_{\mathrm{ae}}$, a trend loss ${ℒ}_{t}$, and a distribution loss ${ℒ}_{\mathrm{dis}}$.

The absolute error loss ${ℒ}_{\mathrm{ae}}$ minimizes the discrepancies between the predicted and the real time series within each time window. The Euclidean distance was adopted to quantify the differences between two sequences in a multidimensional space.

Considering the trend variability of a time series significantly affects the prediction performance of the model (Yoon et al., 2019). Since the fluctuation of BOLD signal is highly stochastic, we defined the trend loss function ${ℒ}_{t}$ to represent the fluctuation direction of the signal by using the sign function.

(9) $${ℒ}_{t}\left(y,\hat{y}\right)=\left|\mathrm{sign}\left({\hat{y}}_{T+1}-{\hat{y}}_{T}\right)-\mathrm{sign}\left(y_{T+1}-y_{T}\right)\right|,$$

where sign denotes the sign function (Lu et al., 2021), which is defined as:

(10) $$\mathrm{sign}\left(x,y\right)=\left\{\begin{array}{cc} 1 & x>y\\ 0 & x=y\\ -1 & x<y \end{array}\right..$$

To enhance the learning ability of the model to the overall characteristics of the historical data, we represented the distribution of historical data via the distribution loss ${ℒ}_{\mathrm{dis}}$. This loss function enables us to examine the statistical characteristics of the distribution of a time series, such as average value and standard deviation of the series window (Yoon et al., 2019).

(11) $${ℒ}_{\mathrm{dis}}\left(y,\hat{y}\right)={\left\|E\left(y\right)-E\left(\hat{y}\right)\right\|}_{1}.$$

By combining ${ℒ}_{g}$ and ${ℒ}_{s}$, the generator loss function could be written as:

(12) $$\begin{array}{l} {ℒ}_{G}\left(y,\hat{y}\right)={ℒ}_{s}\left(y,\hat{y}\right)+{ℒ}_{g}\left(y,\hat{y}\right)\\ \;=\lambda_{\mathrm{ae}}{ℒ}_{\mathrm{ae}}\left(y,\hat{y}\right)+\lambda_{t}{ℒ}_{t}\left(y,\hat{y}\right)+\lambda_{\mathrm{dis}}{ℒ}_{\mathrm{dis}}\left(y,\hat{y}\right)+\lambda_{g}{ℒ}_{g}\left(\hat{y}\right), \end{array}$$

where $\lambda_{\mathrm{ae}}$, $\lambda_{t}$, $\lambda_{\mathrm{dis}}$, and $\lambda_{g}$ are the parameters applied in regulating the absolute error loss, trend loss, distribution loss, and the adversarial loss of generator, respectively.

### 2.7 Test–retest reliability

To assess the clinical significance of our model, we initially constructed the FC matrix by computing Pearson correlations across all time series. Subsequently, we investigated whether the FC of predicted outcomes aligned with ground‐truth values, utilizing the intraclass correlation coefficient (ICC). Originally applied to assess the consistency of functional connectivity across different sessions within the same subject, ICC was adapted in this study to treat prediction results and ground truth as distinct sessions, enabling pre‐ and postprediction retest reliability analyses. Employing the experimental code from (Tozzi et al., 2020), we computed FC matrices for all subjects, yielding an ICC vector wherein each value represented the ICC value at the FC edge group level. We maintained consistency with the ICC grading criteria outlined in (Cicchetti, 1994; Tozzi et al., 2020), distinguishing between poor (<0.40), fair (0.40–0.60), good (0.60–0.75), and excellent (>0.75) agreement. Additionally, we conducted individual‐level comparisons by computing Pearson correlation coefficients between predictions and ground‐truth values.

### 2.8 Classification model

To investigate the clinical application value of the proposed model in brain disorder diagnosis, we conducted a classification analysis based on prolonged signals to examine whether this method could promote the identification of patients with MDD and ASD from the HC.

We performed binary classification experiments on the original and prolonged time series, respectively. A five‐fold cross‐validation was performed in order to prevent any potential bias in selecting individuals for the test set. We used decision tree (Navada et al., 2011), supervised time series forest (Cabello et al., 2020), and some popular ensemble learning models (i.e., the shapelet Transform [Lines et al., 2012], Catch22 [Lubba et al., 2019], and AdaBoost [Mathanker et al., 2011]) as classifiers for classification analysis. We employed the same parameter settings as HCP to train the model and predict future time points, and concatenated the predicted time series and the original time series to achieve a prolonged time series. The lengths of the predicted results were 120 for ASD cohort and 90 for MDD cohort, respectively.

### 2.9 Evaluation metrics

The root mean square error (RMSE) and the mean absolute error (MAE) were used to evaluate the deviation of prediction results from the real sequences, formulated as follows:

(13) $$\text{RMSE}\left(y,\hat{y}\right)=\sqrt{\frac{1}{N}\sum_{i=1}^{N}{\left(y_{i}-{\hat{y}}_{i}\right)}^{2}},$$

(14) $$\mathrm{MAE}\left(y,\hat{y}\right)=\frac{1}{N}\sum_{i=1}^{N}\left|y_{i}-{\hat{y}}_{i}\right|.$$

We also adopted the dynamic time warping (DTW) metric to measure the similarity of shape—an important metric characterizing the global fluctuation information of a sequence—between the two time series (Le Guen & Thome, 2019). Specifically, DTW assesses the sum of the distances between the best matching elements. Given two time series $y=\left[y_{1},\ldots ,y_{i},\ldots ,y_{T}\right]$ and $\hat{y}=\left[{\hat{y}}_{1},\ldots ,{\hat{y}}_{i},\ldots ,{\hat{y}}_{T}\right]$, i and j are the indexes of the time steps of y and $\hat{y}$，respectively; C is a matrix representing the distance between y and $\hat{y}$ element by element. The distance between y and $\hat{y}$ can be calculated as the minimum path of C via dynamic programming, formulated as:

(15) $$\mathrm{DTW}\left(y,\hat{y}\right)=\sum_{\left(i^{\prime },j^{\prime }\right)\in C}\left\|y_{i^{\prime }}-{\hat{y}}_{j^{\prime }}\right\|.$$

We employed five metrics to evaluate the classification performance, including precision, F1‐score, recall score, accuracy, and area under the receiver operator characteristic curve.

## 3 RESULTS

### 3.1 Model implementation

The information of the original time series extracted from subjects of the HCP, ABIDE I‐UM, and REST‐meta‐MDD‐IMH datasets are listed in Table 2. The DMN regions of HCP‐MMP and AAL atlases are given in Tables S2 and S3, respectively. To simplify the prediction task, we utilized the VMD technique to extract IMFs from the original time series. The VMD produced a set of IMFs corresponding to distinct frequency bands. Table 3 presents the detailed frequency range of each IMF.

**TABLE 2:** The characteristics of the time series.

	HCP	ASD	MDD
Length of time series	1200	300	240
Number of variables	16	10	10
Extension length	60	120	90
Sample rate (TR)	0.72 s	2 s	2 s

Abbreviations: ASD, autism spectrum disorder; HCP, Human Connectome Project; MDD, major depressive disorder.

**TABLE 3:** The frequency bands corresponding to each IMF.

	HCP	ASD	MDD
IMF1 (Hz)	0.0117 ± 0.0023	0.0120 ± 0.0001	0.0140 ± 0.0039
IMF2 (Hz)	0.0476 ± 0.0295	0.0287 ± 0.0138	0.0363 ± 0.0140
IMF3 (Hz)	0.2946 ± 0.0810	0.0655 ± 0.0198	0.0641 ± 0.0150
IMF4 (Hz)	0.5199 ± 0.0354	0.0744 ± 0.0178	0.0853 ± 0.0101

*Note*: Values—mean frequency ± mean width/2.

Abbreviations: ASD, autism spectrum disorder; HCP, Human Connectome Project; IMF, intrinsic mode function; MDD, major depressive disorder.

Before feeding the time series data into the FDAA‐Net for prediction, the time series were normalized via the max–min normalization method:

(16) $$\bar{y}=\frac{y-y_{\min}}{y_{\max}-y_{\min}},$$

where y and $\bar{y}$ are the original and normalized data. $y_{\max}$ and $y_{\min}$ are the maximum and minimum oscillation amplitude of the original time series.

For comparison with prior studies, we evaluate the models employed against both contemporary and traditional DL models (i.e., Autoformer [Wu et al., 2021], temporal pattern attention LSTM [TPA‐LSTM; Shih et al., 2019], LSTM [Shastri et al., 2020], and RNN [Wei et al., 2017]) and ML models (Prophet [Taylor & Letham, 2018] and ARIMA [Lazcano et al., 2023]). Baseline details are as shown below. The training process, testing processes, and devices used for baseline models are consistent with the proposed model.

#### 3.1.1 Autoformer

Autoformer is a cutting‐edge neural network architecture that combines the strengths of both transformer‐based models and convolutional neural networks. By incorporating both self‐attention and convolutional layers, Autoformer strikes a balance between capturing global context and local features, making it particularly well‐suited for time series forecasting tasks.

#### 3.1.2 Temporal pattern attention LSTM

TPA‐LSTM combines the strengths of both LSTM networks and attention mechanisms. LSTMs are powerful for capturing long‐term dependencies in sequential data, while attention mechanisms enable the model to focus on relevant parts of the input sequence.

#### 3.1.3 Long short‐term memory

LSTM is a type of RNN architecture specifically designed to address the vanishing gradient problem, which can occur when training traditional RNN. LSTMs are particularly well‐suited for modeling sequential data, such as time series, natural language text, and speech.

#### 3.1.4 Recurrent neural network

RNNs are a class of artificial neural networks designed to process sequential data by maintaining an internal state or memory. Unlike feedforward neural networks, which process input data in a single pass, RNN have connections that form directed cycles, allowing them to exhibit temporal dynamics and capture dependencies across time steps.

#### 3.1.5 Prophet

Prophet is an open‐source forecasting tool developed by Facebook's Core Data Science team. It is specifically designed for time series forecasting tasks and is particularly useful for applications where the data may have multiple seasonality components, historical trends, and holiday effects.

#### 3.1.6 Autoregressive integrated moving average

ARIMA is a widely used statistical method for time series forecasting. It is a class of models that captures different aspects of time series data, including trends, seasonality, and irregularities.

The normalized fMRI time series data were randomly divided into training, validation, and testing sets, following a ratio of 7:2:1 (Kannangara et al., 2018). The training and validation sets were used for learning the in‐depth features of the data and selecting models with optimal generalization performance, respectively; and the testing set was used for the evaluation of the prediction performance. The prediction was performed on the DMN regions of each individual, focusing on the IMF 1 and IMF 2 components within low frequency bands. The final prediction results were the superposition of the predicted signals in IMF 1 and IMF 2 bands.

The inverse time decay method (Liu & Ma, 2022) was used with an initial learning rate set to 0.01. The loss functions $\lambda_{\mathrm{ae}}$, $\lambda_{t}$, $\lambda_{\mathrm{dis}}$, and $\lambda_{g}$ were set to 50, 3, 1, and 1, respectively. We divided the training process into a supervised training phase that only used $L_{s}$ and a joint training phase based on ${ℒ}_{s}$ and ${ℒ}_{g}$. The Adam optimizer was used to train the loss functions. The batch size and nodes of fully‐connected layers were set to 128 and 96, respectively. The experimental results for hyperparameter set selection are given in Tables S4–S9. FC and ICC analyses were performed in Matlab R2020a. Other experiments were implemented through the TensorFlow or PyTorch framework and were trained on CPU (Intel Xeon Cascade Lake 6248) and GPU clusters (Nvidia Nvlink Tesla A100).

### 3.2 Experiments of time series prediction

The performance for time series prediction of our model was assessed based on the HCP dataset. Table 4 summarizes the forecasting results of DMN signals within IMF 1 and IMF 2 bands. We conducted significance tests comparing the predictions of our models with baseline models. Detailed results for each DMN region are listed in Tables S10–S15.

**TABLE 4:** The forecasting results for different IMFs of DMN signals.

	Metric	Proposed	Autoformer	TPA‐LSTM	LSTM	RNN	Prophet	ARIMA
IMF 1	RMSE	**0.0425 ± 0.0158**	0.0513 ± 0.0161	0.0523 ± 0.0114	0.0891 ± 0.0219	0.0894 ± 0.0275	0.3229 ± 0.0446	0.2270 ± 0.0358
	MAE	**0.0343 ± 0.0130**	0.0430 ± 0.0139	0.0433 ± 0.0102	0.0696 ± 0.0195	0.0689 ± 0.0232	0.2835 ± 0.0433	0.1893 ± 0.0299
	DTW	**0.9173 ± 0.3666**	1.2069 ± 0.4373	1.2694 ± 0.2865	2.1964 ± 0.8264	2.2023 ± 1.0723	15.9220 ± 2.5865	10.7267 ± 1.6987
IMF 2	RMSE	**0.1015 ± 0.0211**	0.1091 ± 0.0243	0.1076 ± 0.0231	0.1379 ± 0.0217	0.1636 ± 0.2405	0.2147 ± 0.0353	0.2211 ± 0.0392
	MAE	**0.0816 ± 0.0179**	0.0890 ± 0.0214	0.0881 ± 0.0187	0.1130 ± 0.0185	0.1201 ± 0.0758	0.1821 ± 0.0329	0.1840 ± 0.0337
	DTW	**2.5341 ± 0.7708**	2.9770 ± 0.8850	2.9228 ± 0.7553	4.9231 ± 1.2524	5.2573 ± 2.3060	10.1488 ± 1.9041	10.4250 ± 1.8376
All	RMSE	**0.0720 ± 0.0185**	0.0802 ± 0.0202	0.0800 ± 0.0173	0.1135 ± 0.0218	0.1265 ± 0.1340	0.2688 ± 0.0400	0.2241 ± 0.0375
	MAE	**0.0580 ± 0.0155**	0.0660 ± 0.0176	0.0657 ± 0.0145	0.0913 ± 0.0190	0.0945 ± 0.0495	0.2328 ± 0.0381	0.1867 ± 0.0318
	DTW	**1.7257 ± 0.5687**	2.0919 ± 0.6612	2.0961 ± 0.5209	3.5598 ± 1.0394	3.7298 ± 1.6892	13.0354 ± 2.2453	10.5759 ± 1.7682

*Note*: Bold values indicate the best performance among different methods.

Abbreviations: ARIMA, autoregressive integrated moving average; DMN, default mode network; DTW, dynamic time warping; IMF, intrinsic mode function; LSTM, long short‐term memory; MAE, mean absolute error; RMSE, root mean square error; RNN, recurrent neural network; TPA, temporal pattern attention.

As given in Table 4, the FDAA‐Net performed the best in predicting the BOLD signals within IMF 1 band for most of the DMN regions (also see Tables S10–S15), with the average RMSE, MAE, and DTW of 0.0425, 0.0343, and 0.9173, respectively, outperforming other frequently used methods (i.e., Autoformer, TPA‐LSTM, LSTM, RNN, Prophet, and ARIMA). The second‐best performances were achieved by the Autoformer and TPA‐LSTM. However, they neither integrated the features of time and space nor considered the oscillation direction and shape of the signal, thereby could not surpass the proposed model. We also showed that the DL models (TPA‐LSTM, LSTM, and RNN) performed much better than the traditional ML models (Prophet and ARIMA), suggesting the capability of DL models to capture the features of fMRI signals. Furthermore, the unsatisfactory performance of Prophet and ARIMA could be attributed to the fact that these ML models were originally designed for stationary time series, thereby lacking the capability to effectively model non‐stationary time series with strong nonlinearity and volatility.

The FDAA‐Net remained the best prediction performance relative to other reference models in IMF 2, with the RMSE, MAE, and DTW of 0.1015, 0.0816, and 2.5341, respectively (Table 4), suggesting its well adaption to fMRI time series with different volatility. Similar to the prediction results of IMF 1, the DL models outperformed the ML models in all evaluation metrics. In addition, the prediction performance of IMF 2 was slightly lower than those of IMF 1. We speculated that the increased frequency and wider fluctuations in IMF 2 potentially contribute to its heightened unpredictability.

**FIGURE 6:**
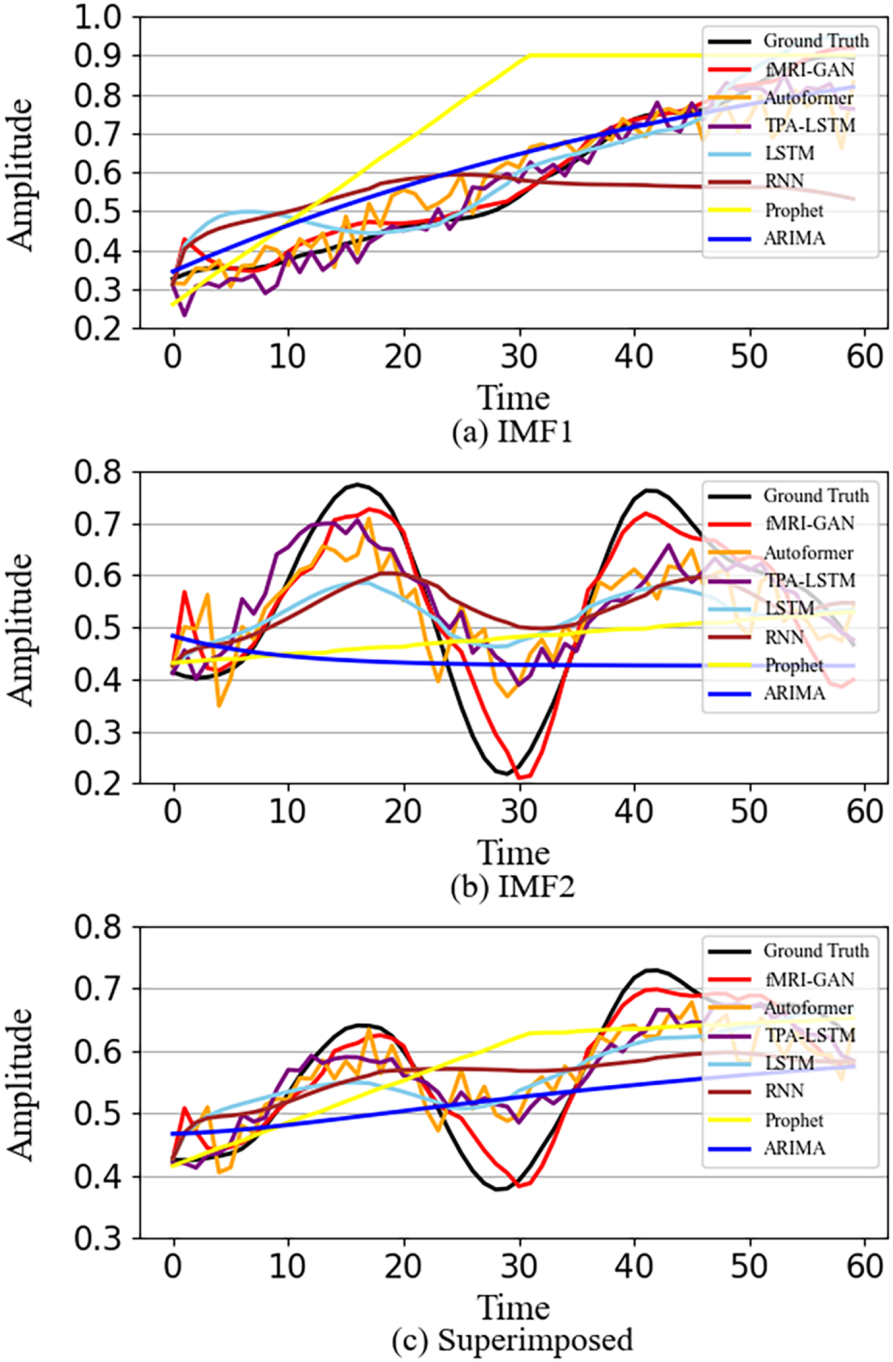
Comparison of the predicted signals in the first default mode network (DMN) region (left medial prefrontal lobe) of a randomly selected subject from the Human Connectome Project (HCP) between different models. The horizontal axis represents the observed time step and the vertical axis stands for the normalized amplitude values. The predicted signal of our model (in red) best fitted to the ground truth (in black) in both shape and fluctuation direction. (a) IMF 1; (b) IMF 2; (c) superimposed. ARIMA, autoregressive integrated moving average; IMF, intrinsic mode function; LSTM, long short‐term memory; RNN, recurrent neural network; TPA, temporal pattern attention.

The visualization depicting the predicted signals is presented in Figure 6, which illustrates the prediction outcomes of IMF 1 for the left medial prefrontal lobe of the DMN in a randomly selected subject. In this visualization, the input consists of the IMF 1 component of the entire DMN region, with the left medial prefrontal lobe as the target region and other DMN regions as auxiliary regions. The output is the prediction of IMF 1 for the left medial prefrontal lobe. The prediction process for IMF 2 follows a similar pattern. Ultimately, the prediction results for IMF 1 and IMF 2 of the left medial prefrontal lobe are combined to form the final prediction outcomes for this subject's left medial prefrontal lobe. These results demonstrate that the proposed model effectively captures the original time series trends with notable flexibility and adaptability. Furthermore, the prediction results for brain regions in other subjects are presented in Figure S2.

### 3.3 Ablation study

To validate the effect of spatiotemporal learning strategy, adversarial training, training phase, and loss function on the prediction result, we performed a variety of ablation experiments on the HCP dataset. The results of the ablation experiments for the combined IMFs are shown in Table 5, and the ablation experiments for each IMF are given in Tables S16 and S17. We showed that removing either distribution or trend loss reduced the prediction performance, suggesting the plausibility of the trend and distribution loss function. The removal of adversarial training largely increased the RMSE and MAE, demonstrating the necessity of learning the global distribution features of the data via adversarial training. Furthermore, the spatial and temporal attention layers enhanced the prediction, which may be explained by the dual attention module adequately captures the strong temporal correlation of fMRI time series and effectively models the robust interactions among brain regions within the DMN during the resting state. These results indicated that all modules and loss functions included in the FDAA‐Net contributed to the improved prediction performance for fMRI time series. In addition, we conducted ablation experiments to assess the impact of predicted length on the prediction accuracy. As shown in Figures S3 and S4, the BOLD signal prediction accuracy was constrained by the length of original time series, and acceptable errors could be achieved with signals having at least 250 time points.

**TABLE 5:** The ablation experiments for each of the main modules.

Ablation strategy	RMSE	MAE	DTW
No trend loss	0.0748 ± 0.0198	0.0595 ± 0.0165	1.7360 ± 0.5928
No distribution loss	0.0739 ± 0.0192	0.0591 ± 0.0159	1.7294 ± 0.6131
No supervised phase	0.0819 ± 0.0211	0.0656 ± 0.0177	2.0569 ± 0.6785
No adversarial phase	0.0832 ± 0.0217	0.0673 ± 0.0182	2.1255 ± 0.7525
No spatial attention layer	0.0761 ± 0.0202	0.0607 ± 0.0168	1.7714 ± 0.6571
No time attention layer	0.0758 ± 0.0198	0.0611 ± 0.0168	1.7927 ± 0.6492
Proposed	**0.0720 ± 0.0185**	**0.0580 ± 0.0155**	**1.7257 ± 0.5687**

*Note*: Values—mean value ± standard deviation. Bold values indicate the best performance among different methods.

Abbreviations: DTW, dynamic time warping; MAE, mean absolute error; RMSE, root mean square error.

### 3.4 Results of test–retest reliability

**FIGURE 7:**
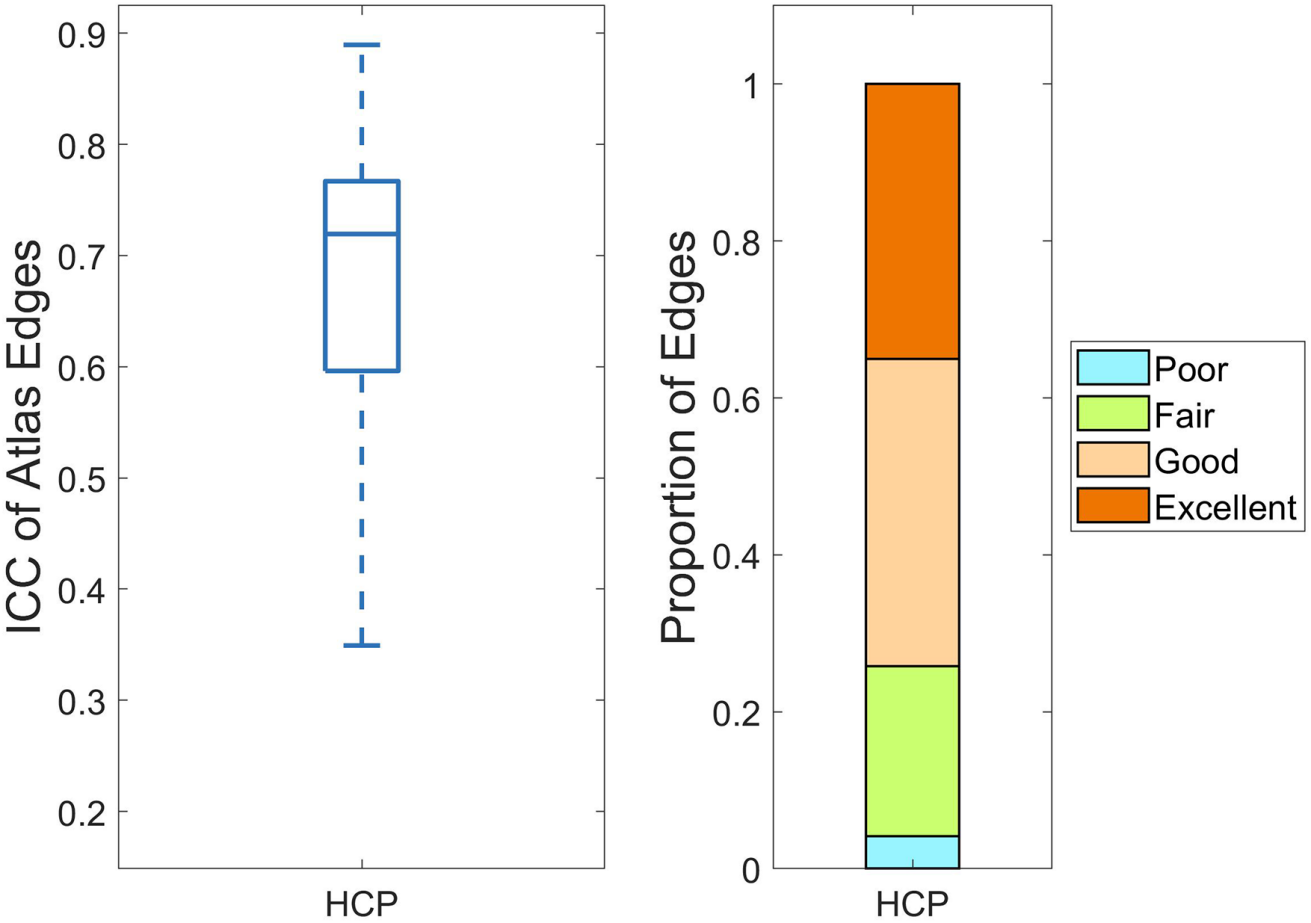
Box plots (left) and grading plots (right) of intraclass correlation coefficient (ICC). The vertical axes both indicate ICC values. HCP, Human Connectome Project.

**FIGURE 8:**
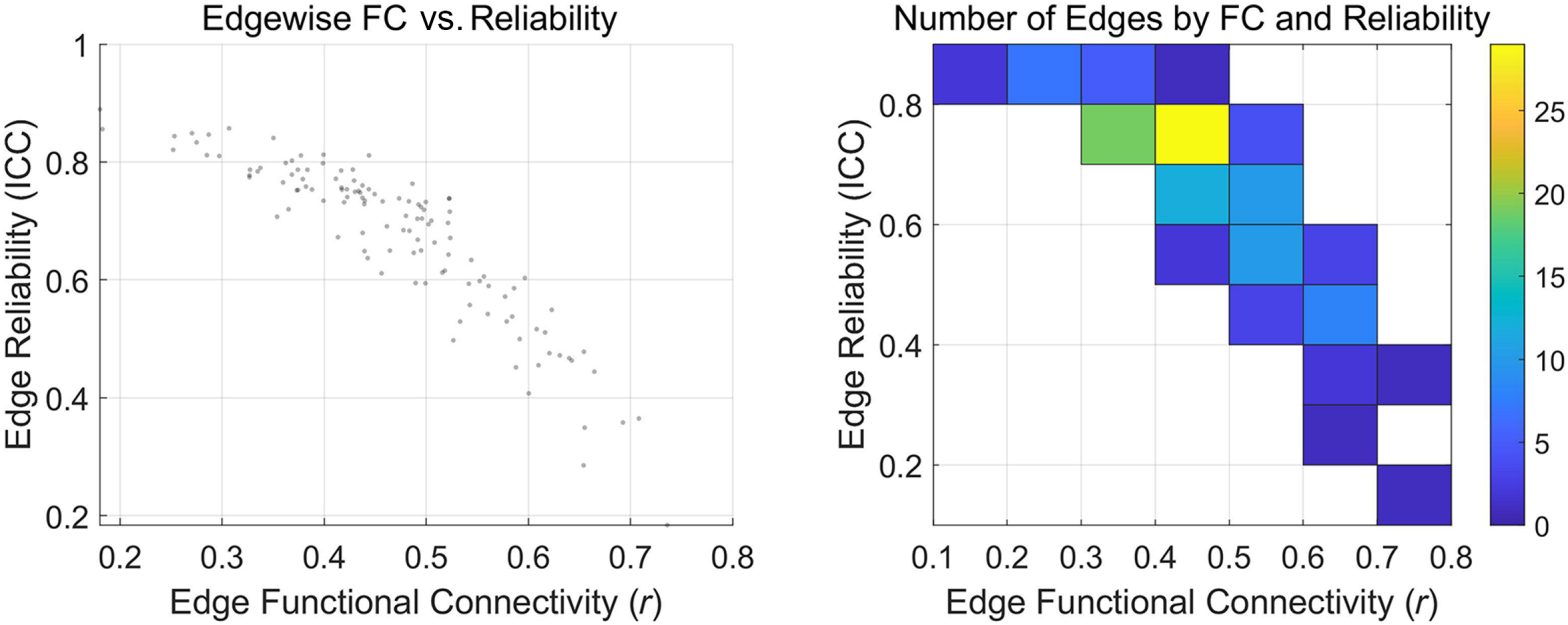
Scatterplot of functional connectivity (FC) edge strengths and intraclass correlation (ICC, left) and distribution of the number of FC edges of different strengths and ICC (right). The vertical axis stands for ICC values and the horizontal axis stands for FC edge strength values. Most of the ICC values in both plots are clustered in 0.6–0.8.

The distribution and grading of the ICC are depicted in Figure 7. The findings reveal that the majority of ICC values fall within the range of 0.6–0.8, corresponding to “Excellent” and “Good” grades. Furthermore, the distribution of FC intensity versus ICC is illustrated in Figure 8. It shows a concentration of ICC values between 0.6 and 0.8, consistent with the observations in Figure 7, indicating a high retest reliability between predictions and targets at the group level. Additionally, Table 6 presents the results of the Pearson correlation coefficient between prediction outcomes and targets, demonstrating a high level of consistency at the individual level. Hence, the proposed method not only achieves precise time‐series prediction but also effectively restores the FC properties of the predicted targets.

**TABLE 6:** Pearson correlation coefficient between predicted outcomes and predicted targets.

DMN ROI ID	Pearson coefficient
ROI1	0.8561 ± 0.1542
ROI2	0.8452 ± 0.2068
ROI3	0.8384 ± 0.1994
ROI4	0.8308 ± 0.2174
ROI5	0.8174 ± 0.2632
ROI6	0.8282 ± 0.2123
ROI7	0.8429 ± 0.1627
ROI8	0.8341 ± 0.2258
ROI9	0.8293 ± 0.2143
ROI10	0.8473 ± 0.2139
ROI11	0.8318 ± 0.2215
ROI12	0.8575 ± 0.1787
ROI13	0.8740 ± 0.1573
ROI14	0.8494 ± 0.2026
ROI15	0.8437 ± 0.1666
ROI16	0.8554 ± 0.1822
Averaged	0.8426 ± 0.2011

*Note*: Values—mean value ± standard deviation.

Abbreviations DMN, default mode network; ROI, region of interest.

### 3.5 Visualization of attention maps

**FIGURE 9:**
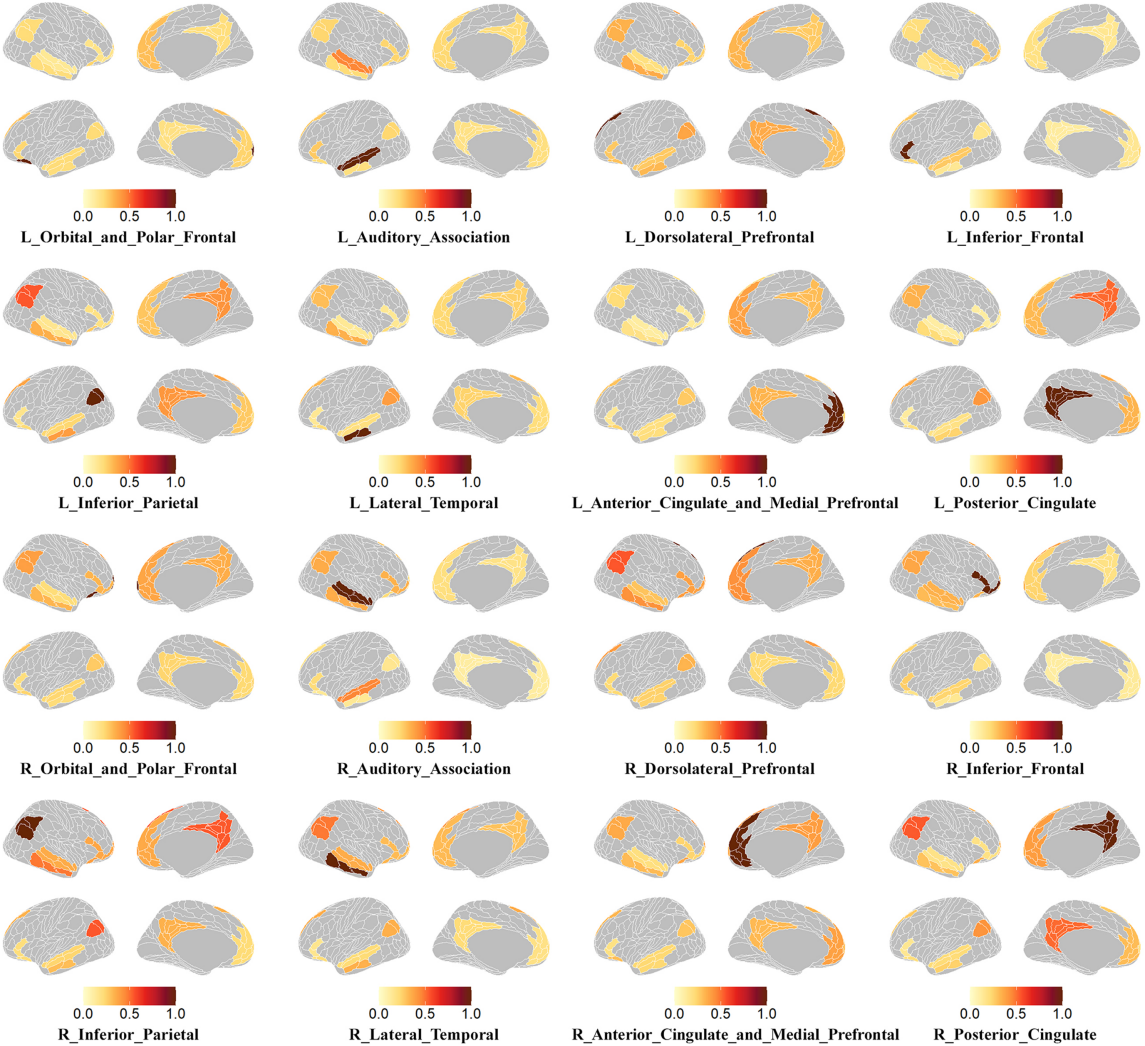
Visualization of attention maps. The darker the color, the greater the contribution of a region.

The contribution of multiple brain regions to the time series prediction of a target region is visualized by mapping the attention values to these regions on the cortical surface (Figure 9) via the ggseg software (Mowinckel & Vidal‐Piñeiro, 2020). Notably, the signal sequence of the contralateral mirrored homotopic region exhibited the highest contribution in most of the prediction tasks. In addition, other regions were also helpful for prediction, such as the right inferior parietal (R_Inferior_Parietal), right posterior cingulate (R_Posterior_Cingulate), right dorsolateral prefrontal (R_Dorsolateral_Prefrontal), and left inferior parietal (L_Inferior_Parietal), which confirmed that the DMN regions are tightly connected. These results are consistent with the current findings indicating interconnections between dorsolateral prefrontal and other brain regions contributed most to the state of consciousness (Long et al., 2016).

### 3.6 Application in brain disease diagnosis

We conducted the diagnostic experiments using data with sufficient fMRI time points (≥250 time points), specifically, 300 for ASD and 250 for MDD, which were the longest time series available in the database. The maximum extension length of the two disease datasets is about 40% of the dataset time point, as given in Table 2. The prediction performances are given in Tables S18–S25. Comparisons of the diagnostic performance between original time series and prolonged time series are given Table 7. We found that, compared with the original time series, the prolonged time series in DMN regions significantly improved the diagnostic power of all the classification models for both MDD and ASD, which achieved the highest diagnostic accuracies of 66.1% and 64.9% for MDD (via Adaboost classifier) and ASD (via Catch22 classifier), respectively. Moreover, the prolonged time series enhanced the classification accuracy of the six classifiers for patients with MDD and ASD by 8.0% and 11.3% on average, respectively. These results demonstrated the adaptability and validity of FDAA‐Net on independent datasets and its potential application value for the diagnostic ability on the existing clinical fMRI data.

**TABLE 7:** Diagnostic performance for patients with MDD and ASD.

	Method	Accuracy	AUC	F1‐score	Recall	Precision
MDD (original)	Decision Tree	0.569 ± 0.103	0.567 ± 0.107	0.508 ± 0.132	0.533 ± 0.172	0.500 ± 0.123
	STSF	0.494 ± 0.141	0.473 ± 0.126	0.288 ± 0.183	0.267 ± 0.162	0.340 ± 0.231
	Shapelet Transform	0.592 ± 0.148	0.570 ± 0.158	0.444 ± 0.284	0.500 ± 0.354	0.417 ± 0.258
	Catch22	0.500 ± 0.111	0.498 ± 0.119	0.459 ± 0.157	0.517 ± 0.207	0.417 ± 0.123
	AdaBoost	0.614 ± 0.149	0.597 ± 0.155	0.443 ± 0.287	0.433 ± 0.276	0.467 ± 0.323
MDD (prolonged)	Decision Tree	**0.592 ± 0.084**	**0.587 ± 0.091**	**0.519 ± 0.128**	0.533 ± 0.172	**0.520 ± 0.113**
	STSF	**0.636 ± 0.083**	**0.613 ± 0.073**	**0.513 ± 0.107**	**0.467 ± 0.172**	**0.700 ± 0.245**
	Shapelet Transform	**0.639 ± 0.159**	**0.640 ± 0.166**	**0.581 ± 0.175**	**0.600 ± 0.255**	**0.623 ± 0.238**
	Catch22	**0.639 ± 0.075**	**0.623 ± 0.051**	**0.568 ± 0.063**	**0.567 ± 0.162**	**0.653 ± 0.185**
	AdaBoost	**0.661 ± 0.154**	**0.642 ± 0.161**	**0.519 ± 0.286**	**0.483 ± 0.260**	**0.567 ± 0.327**
ASD (original)	Decision Tree	0.424 ± 0.117	0.398 ± 0.097	0.506 ± 0.143	0.495 ± 0.185	0.530 ± 0.107
	STSF	0.540 ± 0.090	0.523 ± 0.086	0.614 ± 0.088	0.595 ± 0.131	0.644 ± 0.051
	Shapelet Transform	0.447 ± 0.087	0.419 ± 0.099	0.536 ± 0.107	0.538 ± 0.182	0.573 ± 0.067
	Catch22	0.613 ± 0.173	0.569 ± 0.208	**0.711 ± 0.124**	**0.738 ± 0.102**	0.693 ± 0.163
	AdaBoost	0.504 ± 0.181	0.421 ± 0.163	0.635 ± 0.177	0.743 ± 0.262	0.563 ± 0.121
ASD (prolonged)	Decision Tree	**0.591 ± 0.100**	**0.585 ± 0.108**	**0.653 ± 0.092**	**0.619 ± 0.109**	**0.704 ± 0.104**
	STSF	**0.627 ± 0.093**	**0.632 ± 0.103**	**0.671 ± 0.088**	**0.614 ± 0.125**	**0.763 ± 0.137**
	Shapelet Transform	**0.613 ± 0.060**	**0.577 ± 0.066**	**0.692 ± 0.063**	**0.705 ± 0.129**	**0.693 ± 0.053**
	Catch22	**0.649 ± 0.025**	**0.638 ± 0.041**	0.707 ± 0.021	0.676 ± 0.056	**0.749 ± 0.042**
	AdaBoost	**0.613 ± 0.083**	**0.561 ± 0.079**	**0.710 ± 0.076**	**0.771 ± 0.146**	**0.667 ± 0.046**

*Note*: Values: mean value ± standard deviation. Bold values indicate the best performance among different methods.

Abbreviations: ASD, autism spectrum disorder; AUC, area under the receiver operator characteristic curve; MDD; major depressive disorder; STST, supervised time series forest.

## 4 DISCUSSION

In this study, we proposed an end‐to‐end DL framework, named FDAA‐Net for the prediction of regional BOLD signal sequences. We analyzed the validity of our model in an fMRI time series prediction task and performed ablation analyses of model modules and loss functions. Furthermore, we assessed the test–retest reliability of the predicted results and the FC of the predicted targets, revealing a high level of agreement between the pre‐ and post‐predicted FC. The FDAA‐Net was applied to prolong the BOLD time series of two independent datasets to examine its clinical utility on brain disorder diagnosis. The prolonged BOLD time series exhibited remarkable improvement in diagnostic accuracy. To the best of our knowledge, this is the initial end‐to‐end DL framework designed specifically for forecasting rs‐fMRI time series. Our results demonstrated that the FDAA‐Net could effectively capture the features of historical fMRI data and provide meaningful predictions for the future fluctuation, as well as confirmed the clinical usefulness of our model.

Resting‐state activity is known as the default pattern of brain activity and is defined as a person who is alert and awake and with no active participation in tasks that require attention or have a precise goals‐directed focus (Broyd et al., 2009). The DMN is one of the most active cortical networks during the resting state, its regions demonstrate high co‐activation (strong inter‐regional connectivity), and is highly related to sleep, “wandering”, and self‐referential thinking (Fox et al., 2013). The DMN involves brain regions with strong functional connectivity that are actively engaged during brain rest but turn inactive at the start of a task (Hacker et al., 2013).

Previous studies have reported significant reductions of DMN‐related functional connectivity in patients with ASD (Jung et al., 2014) and MDD (Demirtaş et al., 2016) relative to the HCs, and these connectivity were associated with the clinical phenotype (Hamilton et al., 2011; Jung et al., 2014). Furthermore, studies have also shown remarkable diagnostic value of the DMN on ASD and MDD (Khosla et al., 2019; Scalabrini et al., 2020). These findings suggested that the activity and connectivity of the DMN at rest can be regarded as a valuable biomarker for the diagnosis of some major brain disorders. Therefore, the primary focus of this study was to utilize the BOLD signal sequence of DMN regions for the purpose of developing a time series prediction model, thereby investigating its clinical utility in diagnosing ASD and MDD.

It is a common issue that traditional models fail to satisfy the requirement of precise prediction for highly nonlinear signal solely through constructing local supervised errors limited to a single time step and neglecting of the overall information of the input (Ramadevi & Bingi, 2022). Meanwhile, the GAN has been successful in computer vision and natural language processing (Haidar & Rezagholizadeh, 2019) due to its excellent ability to model the data globally and fully retain the overall information of the input data. Therefore, the proposed method utilized the GAN architecture to learn global information in terms of the distribution and shape of time series. We used a joint training strategy that simultaneously optimized the unsupervised adversarial loss function and supervised loss function. Due to the strong interactions between brain regions within the DMN at the resting state, the BOLD signal of each brain region may change synchronously over time, with very strong correlations in temporal and spatial terms, which is different from other nonlinear time series. Therefore, modeling adequately for the relationships among brain regions and associated characteristics at different time points is essential for fMRI signal prediction. Moreover, traditional models do not adequately capture the spatial–temporal information to model the dynamic action and causality of the underlying process (Cheng et al., 2015), leading to unsatisfied performance. Here, we used the dual attention module to capture such spatiotemporal relationships, which facilitated the model in learning the historical temporal features that were important for characterizing the fMRI signal to be predicted. From the spatial perspective, the dual attention explored the synchronous alteration pattern of fMRI signals and focused on brain regions that showed strong synchronous changes with the target signal. Because the traditional models focused on extracting periodic trend information that cannot be adapted to fMRI data with no obvious periodic variation pattern (Cheng et al., 2015), we introduced a trend loss function to model the fluctuation trend changes of time series in response to the highly volatile nature of fMRI data, which has often been omitted in the GAN model for biological signal prediction (Festag et al., 2022). We speculated that all the aforementioned factors might facilitate the prediction performance of the proposed model.

The “Decomposition and Ensemble”, a hybrid approach that decomposes a signal into a set of independent components, has been introduced to time series forecasting studies (Qiu et al., 2017). The main benefit of this technology is to simplify models, e.g., split a challenging prediction task into several processible subtasks which represent fluctuations of signals in different frequency bands. VMD (Dragomiretskiy & Zosso, 2013), as a decomposition method, has been widely used for decomposing non‐stationary time series predictions, which showed a remarkable ability to improve the performance of time series forecasting when combined with classical ML and DL models (Lahmiri, 2016; Rayi et al., 2022). Previous studies have demonstrated distinct BOLD signal oscillation in different frequency bands (Yuen et al., 2019). Therefore, we proposed VMD to simplify the forecasting model of the BOLD signal while capturing the frequency‐dependent nonlinear oscillation mode of the BOLD signal.

Accumulating evidence has suggested that rs‐fMRI signals in the DMN exhibit evident frequency‐dependent characteristics (Wu et al., 2008). For example, the spectral distribution of the connectivity strength of each brain region within the DMN peaked in a low‐frequency band (<0.05 Hz) (Wu et al., 2008) and the power decreased with increasing frequency (Baria et al., 2011); while cortical regional homogeneity was mainly attributed to BOLD oscillations at 0.01–0.04 Hz (Song et al., 2014). In general, the signal of DMN at a low‐frequency band is one of the most important components of the rs‐fMRI data. Moreover, the BOLD signal measured in fMRI experiments is a slow and indirect measure of neural activity changes. High frequency may include more rapid physiological noise (e.g., cardiac and respiratory cycles) and artifacts (e.g., head motion), which can confound the interpretation of the BOLD signal variations, such as the lower stability of functional connectivity networks (Wu et al., 2008). Even with pretreatment, it remains challenging to fully eliminate the effects of noise. Therefore, we treated the low‐frequency signals as the main research target.

There are several limitations that can be addressed in future work. First, this study only focused on the low‐frequency components of the DMN regions. Although the DMN represents the most stable and active subsystem in the brain during the resting state, low‐frequency signals within it contain crucial information. Therefore, studies encompassing all brain regions, frequency bands, and task‐based data may offer richer information, particularly beneficial for diagnostic practices. This is the primary limitation that restricts the practical application of our model. Future work will be devoted to extending the model to whole brain scale and on high‐frequency components. Second, our model is fine‐grained and requires separate modeling of each brain region for each subject, which may lead to large computational consumption. In the future study, we hope to investigate a group generalized prediction model combined with individual differences to reduce repetitive modeling and introduce group optimization. Third, achieving high prediction accuracy requires a sufficient number of time points in the original fMRI data (at least 250 time points). This constraint the sample size for classification analysis. Therefore, we selected two datasets from the ABIDE and REST‐meta‐MDD databases that met this criterion, with 300 time points for ASD and 250 time points for MDD. Future efforts will aim to address these limitations by developing more efficient network architecture and refining loss functions to accommodate datasets with fewer time points, thereby enhancing generalizability. Fourth, this study primarily investigates whether the time‐series prediction method enhances classification performance and does not specifically optimize the classification model. Subsequent research will focus on tailoring the classification model to leverage timing prediction methods, aiming to achieve superior classification performance.

## 5 CONCLUSION

In this study, we proposed a novel DL framework for forecasting rs‐fMRI time series to address the problem caused by the constraint of collection time of current fMRI scans. As far as we know, this is the first study to predict long‐term fMRI signals using a DL approach. We validated the model on several independent datasets and compared the prediction performance with the conventional methods. Experimental results confirm the efficacy of the proposed method in accurately predicting BOLD signals across all regions within DMN during the resting state while preserving FC properties. We also applied the well‐trained model to assist the diagnosis of brain disorders. The results showed improved diagnostic performance when combining the original time series with the predicted signals, demonstrating the prospective clinical application value of the developed model in enhancing the diagnosis performance of brain disorders through the utilization of existing fMRI data with short scan duration.

## AUTHOR CONTRIBUTIONS

**Weihao Zheng:** Conceptualization; writing—original draft; review and editing; funding acquisition. **Cong Bao:** Methodology; investigation; formal analysis; data curation; writing—original draft; visualization. **Renhui Mu:** Compensatory experiments; software; validation. **Jun Wang:** review and editing; visualization. **Tongtong Li:** Validation; data curation. **Ziyang Zhao:** Visualization; writing—review and editing. **Zhijun Yao:** Conceptualization; writing—review and editing; supervision; funding acquisition. **Bin Hu:** Review and editing; supervision; funding acquisition.

## FUNDING INFORMATION

This work was supported by the Science and Technology Innovation 2030‐Major Projects (2021ZD0202002, 2021ZD0200800, and 2021ZD0200701), the National Natural Science Foundation of China (62227807, 62202212, U21A20520), and the Science and Technology Program of Gansu Province (23YFGA0004).

## CONFLICT OF INTEREST STATEMENT

The authors declare no conflicts of interest.

## Supporting information


**Data S1:** Supplementary information.

## Data Availability

The Human Connectome Project (https://wiki.humanconnectome.org/; Glasser, Smith, et al., 2016), the Autism Brain Imaging Data Exchange I (http://fcon_1000.projects.nitrc.org/indi/abide/abide_I.html) and the REST‐meta‐MDD dataset (http://rfmri.org/REST-meta-MDD) used in this study are publicly available to anyone.
