# Supplementary material for: Frequency‐specific dual‐attention based adversarial network for blood oxygen level‐dependent time series prediction
Source: Hum Brain Mapp. 2024 Sep 27;45(14):e70032. doi: 10.1002/hbm.70032 (PMC11428273; doi:10.1002/hbm.70032)
Supplement: Supplementary file 1 — Data S1: Supplementary information. [file HBM-45-e70032-s001.docx]

**Supplementary Materials**

1. Experiments of the number of IMFs

**Table S1 Evaluation experiments of the number of IMFs**

| HCP | K = 2 | K = 3 | K = 4 | K = 5 | K = 6 |
| --- | --- | --- | --- | --- | --- |
| reconstruction RMSE | 0.1226±0.0128 | 0.0992±0.0147 | **0.0971±0.0130** | 0.0996±0.0128 | 0.1034±0.0122 |
| reconstruction MAE | 0.0975±0.0106 | 0.0788±0.0120 | **0.0772±0.0106** | 0.0791±0.0104 | 0.0822±0.0099 |
| spectral power percentage | 0.9285±0.0168 | 0.9248±0.0172 | **0.9443±0.0131** | 0.9434±0.0133 | 0.9422±0.0135 |
|  |  |  |  |  |  |
| ASD | K = 2 | K = 3 | K = 4 | K = 5 | K = 6 |
| reconstruction RMSE | 0.1434±0.0256 | 0.1123±0.0225 | **0.1115±0.0213** | 0.1153±0.0215 | 0.1185±0.0220 |
| reconstruction MAE | 0.1105±0.0246 | 0.0869±0.0203 | **0.0860±0.0197** | 0.0891±0.0201 | 0.0915±0.0206 |
| spectral power percentage | 0.9179±0.0375 | 0.9379±0.0291 | **0.9399±0.0281** | 0.9386±0.0289 | 0.9375±0.0294 |
|  |  |  |  |  |  |
| MDD | K = 2 | K = 3 | K = 4 | K = 5 | K = 6 |
| reconstruction RMSE | 0.1718±0.0173 | 0.1411±0.0189 | **0.1363±0.0168** | 0.1391±0.0162 | 0.1419±0.0167 |
| reconstruction MAE | 0.1369±0.0163 | 0.1125±0.0166 | **0.1085±0.0146** | 0.1108±0.0143 | 0.1131±0.0148 |
| spectral power percentage | 0.8772±0.0307 | 0.9008±0.0277 | **0.9076±0.0248** | 0.9059±0.0255 | 0.9052±0.0254 |

Note: K means the number of components obtained by the VMD method. The optimal K value is selected by reconstructing low-frequency components to the original sequence and calculating the error.

1. Name of each ROI

**Table S2 The name of ROI of combination results for HCP datasets**

| Serial number | Name |
| --- | --- |
| 1 | L_ Medial_Prefrontal |
| 2 | L_Auditory_Association |
| 3 | L_Dorsolateral_Prefrontal |
| 4 | L_Inferior_Frontal |
| 5 | L_Inferior_Parietal |
| 6 | L_Lateral_Temporal |
| 7 | L_Orbital_and_Polar_Frontal |
| 8 | L_Posterior_Cingulate |
| 9 | R_ Medial_Prefrontal |
| 10 | R_Auditory_Association |
| 11 | R_Dorsolateral_Prefrontal |
| 12 | R_Inferior_Frontal |
| 13 | R_Inferior_Parietal |
| 14 | R_Lateral_Temporal |
| 15 | R_Orbital_and_Polar_Frontal |
| 16 | R_Posterior_Cingulate |

Note: The prefix L denotes the left hemisphere and R denotes the right hemisphere.

**Table S3 The name of each ROI of combination results for ASD and MDD datasets**

| Serial number | Name |
| --- | --- |
| 1 | Superior frontal gyrus, dorsolateral |
| 2 | Superior frontal gyrus, orbital part |
| 3 | Middle frontal gyrus, orbital part |
| 4 | Superior frontal gyrus, medial |
| 5 | Superior frontal gyrus, medial orbital |
| 6 | Anterior cingulate and paracingulate gyri |
| 7 | Median cingulate and paracingulate gyri |
| 8 | Posterior cingulate gyrus |
| 9 | Angular gyrus |
| 10 | Precuneus |

1. Hyper parameters selection experiments

**Table S4 Hyper parameters selection experiments of hidden dim about IMF 1**

| hidden dim | 48 | 96 | 144 |
| --- | --- | --- | --- |
| RMSE | 0.0511±0.0141 | 0.0425±0.0158 | **0.0399±0.0148** |
| MAE | 0.0405±0.0119 | 0.0343±0.0130 | **0.0315±0.0123** |
| DTW | 1.0685±0.3088 | 0.9173±0.3666 | **0.8393±0.3426** |

**Table S5 Hyper parameters selection experiments of batch size about IMF 1**

| batch size | 64 | 128 | 256 |
| --- | --- | --- | --- |
| RMSE | 0.0456±0.0147 | 0.0425±0.0158 | **0.0426±0.0144** |
| MAE | 0.0372±0.0124 | 0.0343±0.0130 | **0.0340±0.0118** |
| DTW | 0.9716±0.3182 | 0.9173±0.3666 | **0.8872±0.3119** |

**Table S6 Hyper parameters selection experiments of num of layers about IMF 1**

| num of layers | 2 | 3 | 4 |
| --- | --- | --- | --- |
| RMSE | **0.0425±0.0158** | 0.0615±0.0200 | 0.0797±0.0222 |
| MAE | **0.0343±0.0130** | 0.0485±0.0174 | 0.0634±0.0192 |
| DTW | **0.9173±0.3666** | 1.3688±0.4913 | 1.8740±0.5975 |

**Table S7 Hyper parameters selection experiments of hidden dim about IMF 2**

| hidden dim | 48 | 96 | 144 |
| --- | --- | --- | --- |
| RMSE | 0.1115±0.0207 | 0.1015±0.0211 | **0.0962±0.0216** |
| MAE | 0.0904±0.0180 | 0.0816±0.0179 | **0.0773±0.0190** |
| DTW | 2.9070±0.7789 | 2.5341±0.7708 | **2.3788±0.8063** |

**Table S8 Hyper parameters selection experiments of batch size about IMF 2**

| batch size | 64 | 128 | 256 |
| --- | --- | --- | --- |
| RMSE | 0.1036±0.0216 | 0.1015±0.0211 | **0.0999±0.0205** |
| MAE | 0.0833±0.0193 | 0.0816±0.0179 | **0.0806±0.0180** |
| DTW | 2.6135±0.8458 | 2.5341±0.7708 | **2.4563±0.7067** |

**Table S9 Hyper parameters selection experiments of num of layer about IMF 2**

| num of layer | 2 | 3 | 4 |
| --- | --- | --- | --- |
| RMSE | **0.1015±0.0211** | 0.1179±0.0210 | 0.1333±0.0224 |
| MAE | **0.0816±0.0179** | 0.0955±0.0185 | 0.1096±0.0205 |
| DTW | **2.5341±0.7708** | 3.2033±0.8470 | 4.0538±0.9415 |

1. The forecasting results for different brain regions in the DMN

**Table S10** The RMSE forecasting results of IMF 1 for different brain regions in the DMN

| DMN ROI ID | Proposed | Autoformer | TPA-LSTM | LSTM | RNN | Prophet | ARIMA |
| --- | --- | --- | --- | --- | --- | --- | --- |
| ROI1 | 0.0520±0.0168 | **0.0454±0.0094** | 0.0480±0.0075 | 0.0920±0.0303 | 0.0760±0.0152 | 0.3300±0.0400 | 0.2325±0.0337 |
| ROI2 | **0.0380±0.0134** | 0.0524±0.0146 | 0.0540±0.0091 | 0.0760±0.0207 | 0.0780±0.0110 | 0.3333±0.0408 | 0.2240±0.0360 |
| ROI3 | **0.0480±0.0341** | 0.0575±0.0237 | 0.0580±0.0154 | 0.0940±0.0207 | 0.0820±0.0130 | 0.2986±0.0438 | 0.2170±0.0413 |
| ROI4 | **0.0480±0.0172** | 0.0508±0.0114 | 0.0500±0.0075 | 0.0820±0.0130 | 0.0740±0.0114 | 0.3157±0.0360 | 0.2210±0.0364 |
| ROI5 | **0.0380±0.0075** | 0.0469±0.0160 | 0.0500±0.0109 | 0.0840±0.0089 | 0.0820±0.0148 | 0.3043±0.0416 | 0.2215±0.0356 |
| ROI6 | **0.0400±0.0172** | 0.0505±0.0157 | 0.0500±0.0109 | 0.0980±0.0228 | 0.0840±0.0152 | 0.3243±0.0360 | 0.2320±0.0238 |
| ROI7 | **0.0460±0.0150** | 0.0519±0.0152 | 0.0520±0.0104 | 0.0920±0.0179 | 0.1040±0.0434 | 0.3443±0.0395 | 0.2360±0.0319 |
| ROI8 | 0.0480±0.0134 | **0.0452±0.0106** | 0.0480±0.0075 | 0.1000±0.0515 | 0.0900±0.0141 | 0.3429±0.0359 | 0.2405±0.0295 |
| ROI9 | **0.0380±0.0109** | 0.0563±0.0159 | 0.0560±0.0065 | 0.0860±0.0207 | 0.0820±0.0148 | 0.3186±0.0324 | 0.2280±0.0333 |
| ROI10 | **0.0340±0.0091** | 0.0547±0.0096 | 0.0540±0.0207 | 0.0740±0.0134 | 0.0700±0.0071 | 0.3386±0.0491 | 0.2215±0.0360 |
| ROI11 | **0.0500±0.0215** | 0.0517±0.0318 | 0.0520±0.0104 | 0.0980±0.0277 | 0.0960±0.0152 | 0.3057±0.0391 | 0.2235±0.0473 |
| ROI12 | **0.0400±0.0109** | 0.0529±0.0260 | 0.0520±0.0168 | 0.0880±0.0084 | 0.1260±0.0767 | 0.3114±0.0564 | 0.2215±0.0330 |
| ROI13 | **0.0340±0.0091** | 0.0539±0.0186 | 0.0560±0.0130 | 0.0780±0.0205 | 0.0980±0.0303 | 0.2971±0.0559 | 0.2220±0.0369 |
| ROI14 | **0.0420±0.0104** | 0.0579±0.0174 | 0.0600±0.0109 | 0.0960±0.0182 | 0.0980±0.0084 | 0.3386±0.0389 | 0.2320±0.0374 |
| ROI15 | **0.0400±0.0134** | 0.0487±0.0117 | 0.0480±0.0075 | 0.0860±0.0114 | 0.0960±0.0152 | 0.3400±0.0733 | 0.2300±0.0486 |
| ROI16 | **0.0440±0.0142** | 0.0445±0.0104 | 0.0480±0.0075 | 0.1020±0.0130 | 0.0940±0.0230 | 0.3229±0.0395 | 0.2295±0.0287 |
| **Averaged** | **0.0425±0.0158** | 0.0513±0.0161 | 0.0523±0.0114 | 0.0891±0.0219 | 0.0894±0.0275 | 0.3229±0.0446 | 0.2270±0.0358 |
| **P Value** | **\** | 0.0004 | 0.0007 | 0.0000 | 0.0000 | 0.0000 | 0.0000 |

**Table S11** The MAE forecasting results of IMF 1 for different brain regions in the DMN

| DMN ROI ID | Proposed | Autoformer | TPA-LSTM | LSTM | RNN | Prophet | ARIMA |
| --- | --- | --- | --- | --- | --- | --- | --- |
| ROI1 | 0.0440±0.0142 | **0.0387±0.0089** | 0.0420±0.0065 | 0.0740±0.0251 | 0.0580±0.0130 | 0.2933±0.0372 | 0.1950±0.0298 |
| ROI2 | **0.0320±0.0104** | 0.0469±0.0111 | 0.0440±0.0091 | 0.0580±0.0179 | 0.0580±0.0110 | 0.2933±0.0413 | 0.1875±0.0301 |
| ROI3 | **0.0400±0.0271** | 0.0413±0.0206 | 0.0460±0.0130 | 0.0720±0.0192 | 0.0620±0.0130 | 0.2586±0.0406 | 0.1795±0.0333 |
| ROI4 | **0.0380±0.0134** | 0.0425±0.0097 | 0.0420±0.0104 | 0.0640±0.0089 | 0.0580±0.0084 | 0.2800±0.0374 | 0.1845±0.0305 |
| ROI5 | **0.0300±0.0075** | 0.0350±0.0136 | 0.0440±0.0091 | 0.0660±0.0114 | 0.0600±0.0122 | 0.2643±0.0374 | 0.1830±0.0275 |
| ROI6 | **0.0340±0.0091** | 0.0431±0.0135 | 0.0420±0.0104 | 0.0780±0.0228 | 0.0640±0.0152 | 0.2829±0.0355 | 0.1935±0.0187 |
| ROI7 | **0.0380±0.0109** | 0.0434±0.0147 | 0.0420±0.0104 | 0.0720±0.0179 | 0.0840±0.0434 | 0.3057±0.0382 | 0.1965±0.0272 |
| ROI8 | 0.0380±0.0075 | **0.0356±0.0102** | 0.0400±0.0075 | 0.0800±0.0453 | 0.0700±0.0141 | 0.3014±0.0353 | 0.2005±0.0248 |
| ROI9 | **0.0320±0.0104** | 0.0513±0.0075 | 0.0460±0.0065 | 0.0680±0.0179 | 0.0640±0.0114 | 0.2771±0.0304 | 0.1905±0.0274 |
| ROI10 | **0.0260±0.0065** | 0.0449±0.0271 | 0.0440±0.0161 | 0.0560±0.0114 | 0.0540±0.0055 | 0.3000±0.0503 | 0.1850±0.0303 |
| ROI11 | **0.0400±0.0215** | 0.0482±0.0142 | 0.0420±0.0104 | 0.0760±0.0219 | 0.0740±0.0114 | 0.2643±0.0378 | 0.1855±0.0391 |
| ROI12 | **0.0300±0.0109** | 0.0483±0.0219 | 0.0440±0.0142 | 0.0680±0.0084 | 0.0980±0.0593 | 0.2714±0.0555 | 0.1845±0.0291 |
| ROI13 | **0.0280±0.0075** | 0.0402±0.0167 | 0.0460±0.0130 | 0.0600±0.0187 | 0.0760±0.0251 | 0.2586±0.0540 | 0.1850±0.0298 |
| ROI14 | **0.0340±0.0091** | 0.0507±0.0145 | 0.0500±0.0109 | 0.0760±0.0182 | 0.0760±0.0089 | 0.3000±0.0387 | 0.1945±0.0328 |
| ROI15 | **0.0320±0.0104** | 0.0386±0.0082 | 0.0400±0.0075 | 0.0620±0.0084 | 0.0740±0.0114 | 0.3000±0.0688 | 0.1925±0.0395 |
| ROI16 | **0.0320±0.0104** | 0.0389±0.0095 | 0.0380±0.0075 | 0.0840±0.0089 | 0.0720±0.0217 | 0.2857±0.0360 | 0.1920±0.0238 |
| **Averaged** | **0.0343±0.0130** | 0.0430±0.0139 | 0.0433±0.0102 | 0.0696±0.0195 | 0.0689±0.0232 | 0.2835±0.0433 | 0.1893±0.0299 |
| **P Value** | **\** | 0.0002 | 0.0008 | 0.0000 | 0.0000 | 0.0000 | 0.0000 |

**Table S12** The DTW forecasting results of IMF 1 for different brain regions in the DMN

| DMN ROI ID | Proposed | Autoformer | TPA-LSTM | LSTM | RNN | Prophet | ARIMA |
| --- | --- | --- | --- | --- | --- | --- | --- |
| ROI1 | 1.2380±0.4044 | **1.0836±0.2243** | 1.1520±0.1541 | 2.3580±1.0015 | 1.7800±0.4598 | 16.6533±2.1899 | 11.0925±1.6441 |
| ROI2 | **0.8340±0.2240** | 1.4228±0.4553 | 1.3520±0.2882 | 1.8120±0.6547 | 1.7820±0.2773 | 16.5533±2.5046 | 10.5150±1.6958 |
| ROI3 | **1.1340±0.8863** | 1.3785±0.6385 | 1.4340±0.4023 | 2.3840±0.6559 | 1.8640±0.3951 | 14.4200±2.4664 | 10.2115±1.9357 |
| ROI4 | **0.9940±0.3834** | 1.1705±0.4033 | 1.1940±0.2415 | 1.9480±0.3408 | 1.7360±0.5597 | 15.4829±2.0464 | 10.4785±1.7349 |
| ROI5 | **0.7820±0.1578** | 1.2001±0.5795 | 1.2800±0.3650 | 1.9800±0.3971 | 1.9480±0.5427 | 14.6543±2.2534 | 10.3655±1.5595 |
| ROI6 | **0.8640±0.3394** | 0.9206±0.3271 | 1.1420±0.1887 | 2.3880±0.7849 | 1.8880±0.4332 | 15.8186±2.0416 | 10.9535±1.0830 |
| ROI7 | **1.0160±0.3274** | 1.3361±0.3801 | 1.2660±0.2354 | 2.3020±0.4380 | 3.0200±2.1520 | 17.2614±2.2044 | 11.1660±1.5621 |
| ROI8 | 0.9700±0.2394 | **0.9578±0.2074** | 1.1220±0.1229 | 2.8480±2.5365 | 2.1480±0.5016 | 16.8986±1.9570 | 11.3655±1.4193 |
| ROI9 | **0.8360±0.1901** | 1.3812±0.2144 | 1.3060±0.1109 | 2.0500±0.6324 | 1.9140±0.3428 | 15.6657±1.7900 | 10.8365±1.6218 |
| ROI10 | **0.7340±0.1623** | 1.1289±0.7448 | 1.3580±0.4665 | 1.7160±0.3300 | 1.6500±0.2523 | 17.0357±2.9843 | 10.4370±1.7432 |
| ROI11 | **1.1040±0.6643** | 1.3968±0.3623 | 1.2300±0.2082 | 2.4100±0.7112 | 2.3060±0.4175 | 14.8229±2.3184 | 10.5025±2.2071 |
| ROI12 | **0.8180±0.2888** | 1.1563±0.8171 | 1.3260±0.5234 | 2.1000±0.3052 | 3.6900±3.0060 | 15.3729±3.3740 | 10.4780±1.5930 |
| ROI13 | **0.7300±0.1319** | 1.1726±0.5961 | 1.3600±0.3754 | 1.7820±0.5330 | 2.4880±0.8994 | 14.4357±3.1782 | 10.4515±1.7111 |
| ROI14 | **0.9080±0.2038** | 1.536±0.5286 | 1.4560±0.3244 | 2.3320±0.4928 | 2.3620±0.4446 | 16.9257±2.3125 | 11.0015±1.8771 |
| ROI15 | **0.8120±0.2332** | 1.1374±0.2526 | 1.1700±0.1477 | 1.9620±0.3497 | 2.2740±0.4775 | 16.9414±4.1888 | 10.9480±2.2354 |
| ROI16 | **0.9020±0.2918** | 0.9307±0.2653 | 1.1620±0.1495 | 2.7700±0.5136 | 2.3860±0.7822 | 15.8100±2.2573 | 10.8240±1.3144 |
| **Averaged** | **0.9173±0.3666** | 1.2070±0.4373 | 1.2694±0.2865 | 2.1964±0.8264 | 2.2023±1.0723 | 15.9220±2.5865 | 10.7267±1.6987 |
| **P Value** | **\** | 0.0005 | 0.0006 | 0.0000 | 0.0000 | 0.0000 | 0.0000 |

**Table S13** The RMSE forecasting results of IMF 2 for different brain regions in the DMN

| DMN ROI ID | Proposed | Autoformer | TPA-LSTM | LSTM | RNN | Prophet | ARIMA |
| --- | --- | --- | --- | --- | --- | --- | --- |
| ROI1 | **0.1000±0.0271** | 0.1110±0.0298 | 0.1180±0.0250 | 0.1360±0.0167 | 0.1400±0.0100 | 0.1917±0.0172 | 0.2130±0.0412 |
| ROI2 | **0.1060±0.0212** | 0.1181±0.0203 | 0.1180±0.0172 | 0.1440±0.0219 | 0.1440±0.0288 | 0.2133±0.0403 | 0.2165±0.0366 |
| ROI3 | **0.0920±0.0237** | 0.1148±0.0271 | 0.1100±0.0227 | 0.1380±0.0363 | 0.1260±0.0251 | 0.2186±0.0285 | 0.2210±0.0299 |
| ROI4 | **0.1000±0.0134** | 0.1047±0.0192 | 0.0940±0.0161 | 0.1440±0.0230 | 0.1380±0.0228 | 0.2243±0.0364 | 0.2240±0.0370 |
| ROI5 | 0.1060±0.0130 | **0.0874±0.0275** | 0.0880±0.0227 | 0.1280±0.0259 | 0.1340±0.0241 | 0.2400±0.0252 | 0.2485±0.0426 |
| ROI6 | **0.1060±0.0237** | 0.1197±0.0282 | 0.1160±0.0237 | 0.1320±0.0130 | 0.1300±0.0224 | 0.2114±0.0353 | 0.2095±0.0382 |
| ROI7 | **0.1160±0.0297** | 0.1252±0.0251 | 0.1320±0.0212 | 0.1440±0.0241 | 0.1400±0.0235 | 0.1900±0.0115 | 0.1965±0.0333 |
| ROI8 | **0.1020±0.0279** | 0.1122±0.0106 | 0.1140±0.0091 | 0.1420±0.0217 | 0.1460±0.0261 | 0.2100±0.0387 | 0.2310±0.0304 |
| ROI9 | **0.1000±0.0172** | 0.1193±0.0099 | 0.1140±0.0091 | 0.1260±0.0114 | 0.1260±0.0114 | 0.2171±0.0214 | 0.2110±0.0373 |
| ROI10 | **0.0980±0.0134** | 0.1092±0.0239 | 0.1040±0.0207 | 0.1340±0.0114 | 0.1340±0.0114 | 0.2014±0.0570 | 0.2165±0.0453 |
| ROI11 | **0.1040±0.0275** | 0.1116±0.0449 | 0.1160±0.0376 | 0.1380±0.0249 | 0.1460±0.0261 | 0.2271±0.0423 | 0.2300±0.0373 |
| ROI12 | 0.1020±0.0168 | 0.0977±0.0288 | **0.0920±0.0237** | 0.1400±0.0200 | 0.1500±0.0235 | 0.2100±0.0337 | 0.2275±0.0441 |
| ROI13 | **0.1000±0.0172** | 0.1049±0.0188 | 0.1040±0.0161 | 0.1420±0.0148 | 0.5720±0.9550 | 0.2371±0.0457 | 0.2360±0.0445 |
| ROI14 | 0.1000±0.0281 | 0.1020±0.0252 | **0.0960±0.0212** | 0.1340±0.0230 | 0.1380±0.0268 | 0.2171±0.0364 | 0.2235±0.0357 |
| ROI15 | **0.0920±0.0130** | 0.0960±0.0216 | 0.0980±0.0187 | 0.1380±0.0349 | 0.1220±0.0130 | 0.2043±0.0282 | 0.2035±0.0327 |
| ROI16 | **0.1000±0.0290** | 0.1111±0.0271 | 0.1080±0.0227 | 0.1460±0.0305 | 0.1320±0.0192 | 0.2214±0.0344 | 0.2290±0.0371 |
| **Averaged** | **0.1015±0.0211** | 0.1091±0.0243 | 0.1076±0.0231 | 0.1379±0.0217 | 0.1636±0.2405 | 0.2147±0.0353 | 0.2211±0.0392 |
| **P Value** | **\** | 0.0000 | 0.0000 | 0.0000 | 0.0000 | 0.0000 | 0.0000 |

**Table S14** The MAE forecasting results of IMF 2 for different brain regions in the DMN

| DMN ROI ID | Proposed | Autoformer | TPA-LSTM | LSTM | RNN | Prophet | ARIMA |
| --- | --- | --- | --- | --- | --- | --- | --- |
| ROI1 | **0.0800±0.0202** | 0.0941±0.0251 | 0.0980±0.0187 | 0.1100±0.0141 | 0.1140±0.0055 | 0.1600±0.0167 | 0.1770±0.0360 |
| ROI2 | **0.0880±0.0172** | 0.0988±0.0181 | 0.0980±0.0109 | 0.1180±0.0192 | 0.1180±0.0259 | 0.1817±0.0343 | 0.1805±0.0307 |
| ROI3 | **0.0740±0.0207** | 0.0905±0.0246 | 0.0880±0.0202 | 0.1120±0.0311 | 0.1020±0.0217 | 0.1871±0.0243 | 0.1850±0.0252 |
| ROI4 | **0.0780±0.0109** | 0.0876±0.0169 | 0.0800±0.0134 | 0.1200±0.0187 | 0.1140±0.0241 | 0.1929±0.0377 | 0.1870±0.0320 |
| ROI5 | 0.0860±0.0130 | **0.0715±0.0242** | 0.0720±0.0184 | 0.1060±0.0195 | 0.1100±0.0187 | 0.2071±0.0229 | 0.2070±0.0361 |
| ROI6 | **0.0840±0.0178** | 0.0967±0.0238 | 0.0940±0.0161 | 0.1100±0.0100 | 0.1060±0.0152 | 0.1786±0.0339 | 0.1750±0.0338 |
| ROI7 | **0.0980±0.0261** | 0.1016±0.0227 | 0.1060±0.0184 | 0.1180±0.0228 | 0.1180±0.0217 | 0.1571±0.0095 | 0.1615±0.0291 |
| ROI8 | **0.0800±0.0202** | 0.0936±0.0093 | 0.0940±0.0091 | 0.1200±0.0187 | 0.1180±0.0217 | 0.1771±0.0350 | 0.1925±0.0265 |
| ROI9 | **0.0820±0.0150** | 0.0944±0.0089 | 0.0940±0.0091 | 0.1020±0.0130 | 0.1040±0.0114 | 0.1829±0.0198 | 0.1755±0.0328 |
| ROI10 | **0.0800±0.0109** | 0.0896±0.0216 | 0.0860±0.0150 | 0.1060±0.0089 | 0.1120±0.0130 | 0.1686±0.0527 | 0.1790±0.0397 |
| ROI11 | **0.0840±0.0207** | 0.0917±0.0399 | 0.0940±0.0294 | 0.1140±0.0230 | 0.1180±0.0217 | 0.1943±0.0387 | 0.1910±0.0316 |
| ROI12 | 0.0820±0.0168 | 0.0805±0.0249 | **0.0780±0.0187** | 0.1140±0.0182 | 0.1240±0.0207 | 0.1771±0.0315 | 0.1890±0.0363 |
| ROI13 | **0.0800±0.0172** | 0.0848±0.0166 | 0.0840±0.0161 | 0.1180±0.0130 | 0.2480±0.2922 | 0.2071±0.0419 | 0.1970±0.0385 |
| ROI14 | **0.0780±0.0239** | 0.0823±0.0224 | **0.0780±0.0172** | 0.1120±0.0192 | 0.1100±0.0245 | 0.1829±0.0315 | 0.1870±0.0316 |
| ROI15 | **0.0740±0.0120** | 0.0765±0.0191 | 0.0780±0.0187 | 0.1100±0.0235 | 0.0960±0.0089 | 0.1729±0.0275 | 0.1695±0.0274 |
| ROI16 | **0.0780±0.0227** | 0.0902±0.0235 | 0.0880±0.0187 | 0.1180±0.0277 | 0.1100±0.0158 | 0.1857±0.0305 | 0.1905±0.0309 |
| **Averaged** | **0.0816±0.0179** | 0.0890±0.0214 | 0.0881±0.0187 | 0.1130±0.0185 | 0.1201±0.0758 | 0.1821±0.0329 | 0.1840±0.0337 |
| **P Value** | **\** | 0.0000 | 0.0000 | 0.0000 | 0.0000 | 0.0000 | 0.0000 |

**Table S15** The DTW forecasting results of IMF 2 for different brain regions in the DMN

| DMN ROI ID | Proposed | Autoformer | TPA-LSTM | LSTM | RNN | Prophet | ARIMA |
| --- | --- | --- | --- | --- | --- | --- | --- |
| ROI1 | **2.4900±0.7833** | 3.2215±0.9848 | 3.2880±0.7206 | 4.8820±1.4313 | 5.0840±0.5984 | 8.3500±1.9704 | 9.9590±1.7812 |
| ROI2 | **2.7000±0.6737** | 3.3585±1.0128 | 3.5000±0.7414 | 5.5860±0.8764 | 5.4360±1.1959 | 10.2183±2.0341 | 10.2210±1.7306 |
| ROI3 | **2.2580±0.7930** | 2.9417±1.0770 | 2.9660±0.8069 | 5.0720±1.7913 | 4.0100±1.2464 | 10.3600±1.3362 | 10.4245±1.4186 |
| ROI4 | **2.4080±0.3520** | 2.9287±0.9288 | 2.5720±0.6875 | 5.3660±1.0313 | 4.9000±1.3849 | 10.8314±2.1470 | 10.5870±1.7736 |
| ROI5 | 2.6160±0.5209 | **2.0736±0.7235** | 2.2440±0.5365 | 3.9980±0.7352 | 4.4720±0.6642 | 11.5971±1.1039 | 11.6900±1.9696 |
| ROI6 | **2.9160±1.3992** | 3.1082±1.2952 | 3.4080±0.9586 | 5.1020±0.7416 | 4.9160±1.2779 | 10.0100±1.7534 | 9.8815±1.7801 |
| ROI7 | **3.5700±1.3523** | 3.7627±0.8454 | 3.9060±0.6303 | 6.1900±1.6373 | 5.9920±1.3589 | 8.3429±1.4262 | 9.3245±1.5865 |
| ROI8 | **2.3600±0.7181** | 2.7562±0.3955 | 2.9560±0.2850 | 4.7580±1.0587 | 4.7480±1.0117 | 10.0200±1.9302 | 10.9010±1.4854 |
| ROI9 | **2.5060±0.7257** | 3.6083±0.4131 | 3.2100±0.3024 | 4.7180±1.3296 | 4.6340±1.0657 | 10.1886±1.1364 | 9.9280±1.7227 |
| ROI10 | **2.3900±0.3140** | 2.6863±0.7283 | 2.7700±0.5302 | 4.7760±1.2117 | 4.9780±0.8499 | 9.6271±2.8210 | 10.1850±2.1397 |
| ROI11 | **2.4980±0.9145** | 2.8103±1.3650 | 2.9640±1.0215 | 4.7360±1.5948 | 4.9780±1.3261 | 10.8757±2.0419 | 10.8685±1.7515 |
| ROI12 | 2.5840±0.6683 | 2.9212±1.1422 | **2.4500±0.8509** | 4.6180±1.3088 | 4.9800±1.3100 | 9.9200±1.5989 | 10.6905±2.0146 |
| ROI13 | **2.3340±0.6130** | 2.5710±0.8747 | 2.7160±0.6490 | 4.6880±0.8002 | 12.1700±15.8845 | 11.6000±2.3034 | 11.1545±2.1285 |
| ROI14 | **2.4100±0.6171** | 2.7049±0.8336 | 2.4500±0.6251 | 4.3720±0.9706 | 4.4800±1.1202 | 10.2843±1.7490 | 10.5995±1.7459 |
| ROI15 | **2.1520±0.2769** | 3.0253±0.7236 | 2.5140±0.5335 | 4.6800±1.2744 | 3.8940±0.7033 | 9.7057±1.3617 | 9.5930±1.6010 |
| ROI16 | **2.3540±0.7340** | 3.1540±0.8166 | 2.8500±0.5935 | 5.2280±1.8563 | 4.4440±1.1455 | 10.4500±1.6142 | 10.7925±1.7049 |
| **Averaged** | **2.5341±0.7708** | 2.9770±0.8850 | 2.9228±0.7553 | 4.9231±1.2524 | 5.2573±2.3060 | 10.1488±1.9041 | 10.4250±1.8376 |
| **P Value** | **\** | 0.0000 | 0.0000 | 0.0000 | 0.0000 | 0.0000 | 0.0000 |

**Table S16** Ablation experiments for each of the main modules for IMF 1

|  | RMSE | MAE | DTW |
| --- | --- | --- | --- |
| no trend loss | 0.0458±0.0169 | 0.0357±0.0136 | 1.4041±0.3766 |
| no distribution loss | 0.0455±0.0163 | 0.0355±0.0133 | 1.4022±0.3735 |
| no supervised phase | 0.0511±0.0178 | 0.0402±0.0155 | 1.5977±0.3939 |
| no adversarial phase | 0.0525±0.0179 | 0.0416±0.0151 | 1.6420±0.4201 |
| no spatial attention layer | 0.0466±0.0170 | 0.0366±0.0138 | 1.4096±0.3798 |
| no time attention layer | 0.0470±0.0174 | 0.0377±0.0147 | 1.5083±0.3807 |
| proposed | **0.0425±0.0158** | **0.0343±0.0130** | **0.9173±0.3666** |

**Table S17** Ablation experiments for each of the main modules for IMF 2

|  | RMSE | MAE | DTW |
| --- | --- | --- | --- |
| no trend loss | 0.1038±0.0227 | 0.0832±0.0194 | 2.5679±0.8089 |
| no distribution loss | 0.1023±0.0221 | 0.0826±0.0185 | 2.5365±0.8526 |
| no supervised phase | 0.1127±0.0243 | 0.0909±0.0198 | 3.0160±0.9630 |
| no adversarial phase | 0.1138±0.0254 | 0.0929±0.0213 | 3.1089±1.0849 |
| no spatial attention layer | 0.1056±0.0234 | 0.0848±0.0197 | 2.6332±0.9344 |
| no time attention layer | 0.1045±0.0221 | 0.0844±0.0188 | 2.5771±0.9177 |
| proposed | **0.1015±0.0211** | **0.0816±0.0179** | **2.5341±0.7708** |

1. Experiments of the length of the model inputs and outputs for disease datasets

**Table S18 Evaluation of the different lengths of the model inputs and outputs for disease group of ASD dataset of IMF 1**

|  | 120 | 90 | 60 |
| --- | --- | --- | --- |
| RMSE | **0.0193±0.0122** | 0.0245±0.0132 | 0.0269±0.0152 |
| MAE | **0.0134±0.0080** | 0.0163±0.0094 | 0.0192±0.0114 |
| DTW | 1.0464±0.5813 | 1.0008±0.5167 | **0.7388±0.3730** |

**Table S19 Evaluation of the different lengths of the model inputs and outputs for the healthy group of ASD dataset of IMF 1**

|  | 120 | 90 | 60 |
| --- | --- | --- | --- |
| RMSE | 0.0196±0.0132 | 0.0256±0.0140 | **0.0186±0.0126** |
| MAE | 0.0130±0.0087 | 0.0167±0.0099 | **0.0123±0.0073** |
| DTW | 1.0507±0.5958 | 1.0176±0.5400 | **0.9358±0.5038** |

**Table S20 Evaluation of the different lengths of the model inputs and outputs for the disease group of MDD dataset of IMF 1**

|  | 90 | 70 | 50 |
| --- | --- | --- | --- |
| RMSE | **0.0209±0.0103** | 0.0212±0.0146 | 0.0283±0.0129 |
| MAE | **0.0139±0.0066** | 0.0143±0.0106 | 0.0197±0.0101 |
| DTW | 0.9275±0.3614 | 0.7262±0.4667 | **0.7011±0.2839** |

**Table S21 Evaluation of the different lengths of the model inputs and outputs for the healthy group of MDD dataset of IMF 1**

|  | 90 | 70 | 50 |
| --- | --- | --- | --- |
| RMSE | 0.0218±0.0158 | 0.0251±0.0129 | **0.0174±0.0109** |
| MAE | 0.0144±0.0090 | 0.0164±0.0094 | **0.0123±0.0075** |
| DTW | 0.9777±0.4900 | 0.8707±0.3800 | **0.7833±0.2871** |

**Table S22 Evaluation of the different lengths of the model inputs and outputs for the disease group of ASD dataset of IMF 2**

|  | 120 | 90 | 60 |
| --- | --- | --- | --- |
| RMSE | **0.0357±0.0179** | 0.0422±0.0179 | 0.0485±0.0189 |
| MAE | **0.0219±0.0122** | 0.0273±0.0126 | 0.0330±0.0154 |
| DTW | 1.9561±1.0835 | 1.7517±0.7161 | **1.4150±0.6155** |

**Table S23 Evaluation of the different lengths of the model inputs and outputs for the healthy group of ASD dataset of IMF 2**

|  | 120 | 90 | 60 |
| --- | --- | --- | --- |
| RMSE | 0.0351±0.0168 | 0.0420±0.0177 | **0.0338±0.0131** |
| MAE | 0.0212±0.0111 | 0.0269±0.0130 | **0.0114±0.0092** |
| DTW | 1.8853±0.8444 | 1.7421±0.7358 | **1.5962±0.6815** |

**Table S24 Evaluation of the different lengths of the model inputs and outputs for the disease group of MDD dataset of IMF 2**

|  | 90 | 70 | 50 |
| --- | --- | --- | --- |
| RMSE | 0.0407±0.0192 | **0.0340±0.0190** | 0.0524±0.0201 |
| MAE | 0.0238±0.0118 | **0.0219±0.0121** | 0.0352±0.0155 |
| DTW | 1.7170±0.8204 | **1.2136±0.6589** | 1.3474±0.5379 |

**Table S25 Evaluation of the different lengths of the model inputs and outputs for the healthy group of MDD dataset of IMF 2**

|  | 90 | 70 | 50 |
| --- | --- | --- | --- |
| RMSE | 0.0394±0.0187 | 0.0407±0.0171 | **0.0363±0.0158** |
| MAE | 0.0234±0.0122 | 0.0262±0.0121 | **0.0192±0.0085** |
| DTW | 1.6771±0.7448 | 1.4768±0.6223 | **1.2407±0.5661** |

1. Hyper parameters selection experiments

From Table S4 to Table S9, the best parameters are batch size=256, hidden dim=144 and num of layers=2. However, batch size=256 and hidden dim=144 will run 2-3 times longer than batch size=128 and hidden dim=96 with little difference. Therefore, we set batch size=128, hidden dim=96 and num of layer =2.

1. Comparison of ground truth and predicted results

From Fig. S2, we can find that whether the predicted results of the components or the combined predicted results of the components, the predicted values are very close to the actual values. And in comparison with the original series, the model can predict the direction of the series trend.

1. Experiments of the length of the model inputs and outputs for disease datasets

As can be seen from Table S18 to Table S25, the prediction errors from the inputs and outputs of the different models are within acceptable limits. Therefore, we choose the longest model output length parameters as the final parameters.

1. Experiments of the number of IMFs


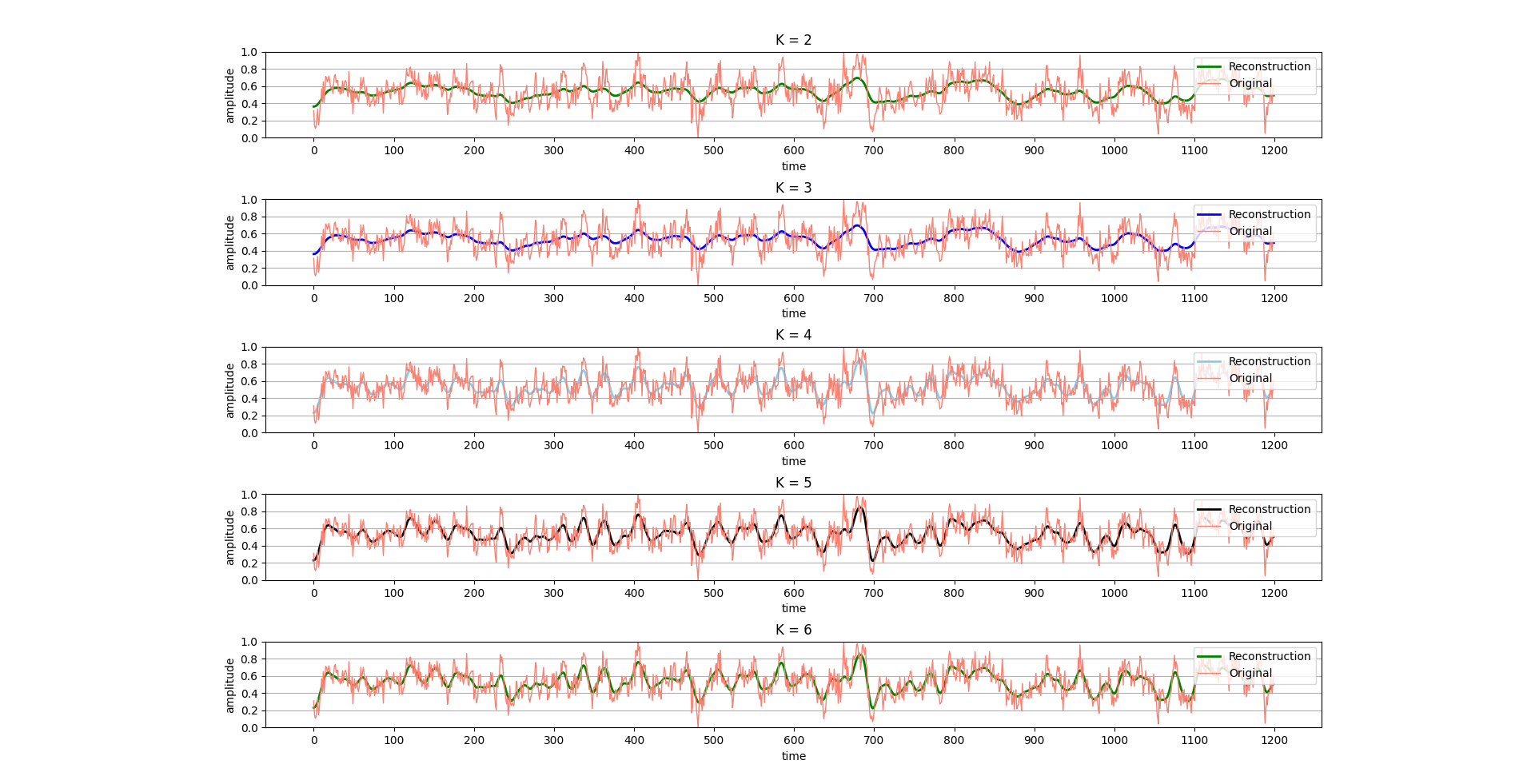


**Fig. S1. The reconstruction time series about different K.**

1. Comparison of ground truth and predicted results

**
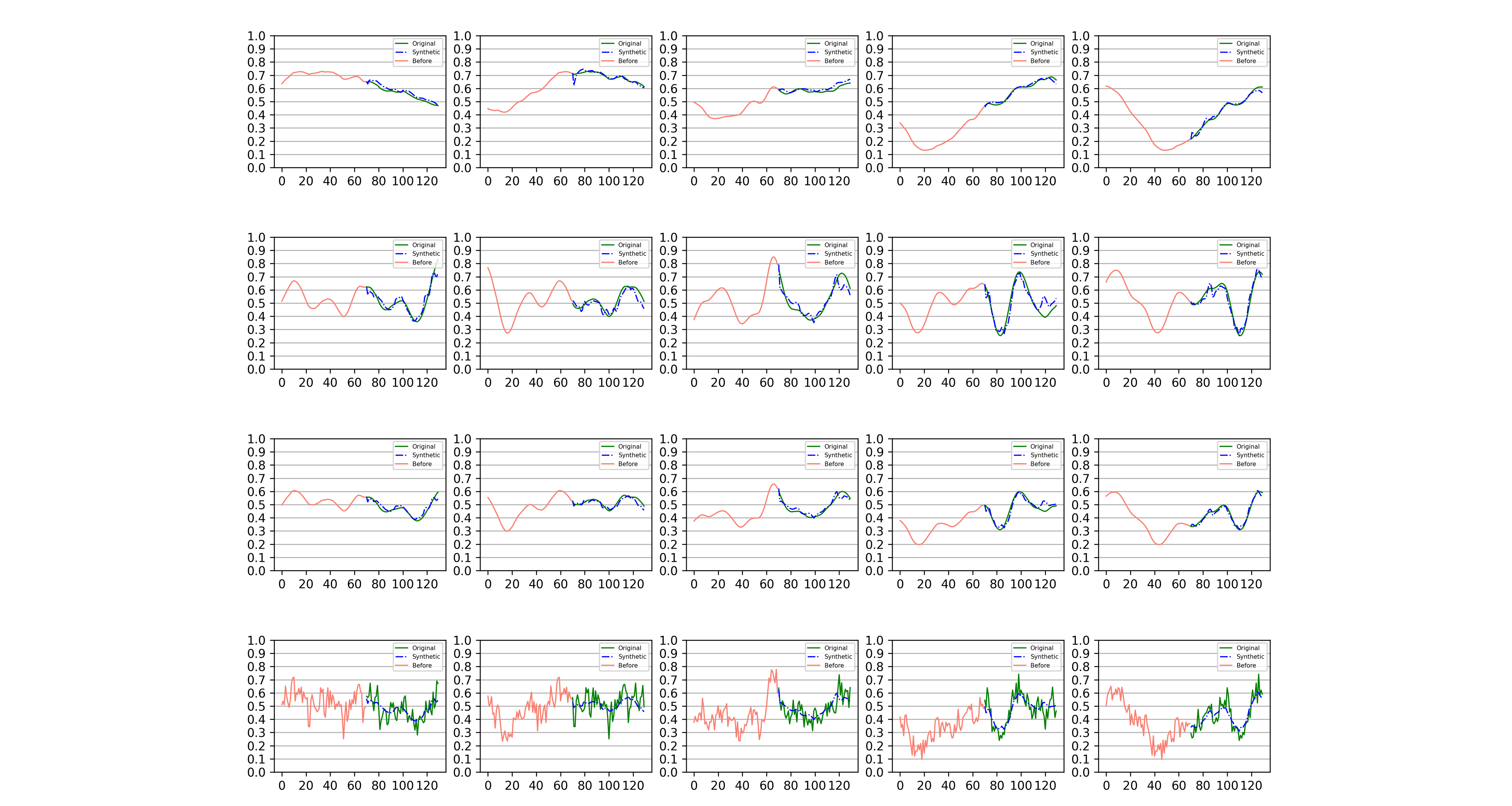
**

**
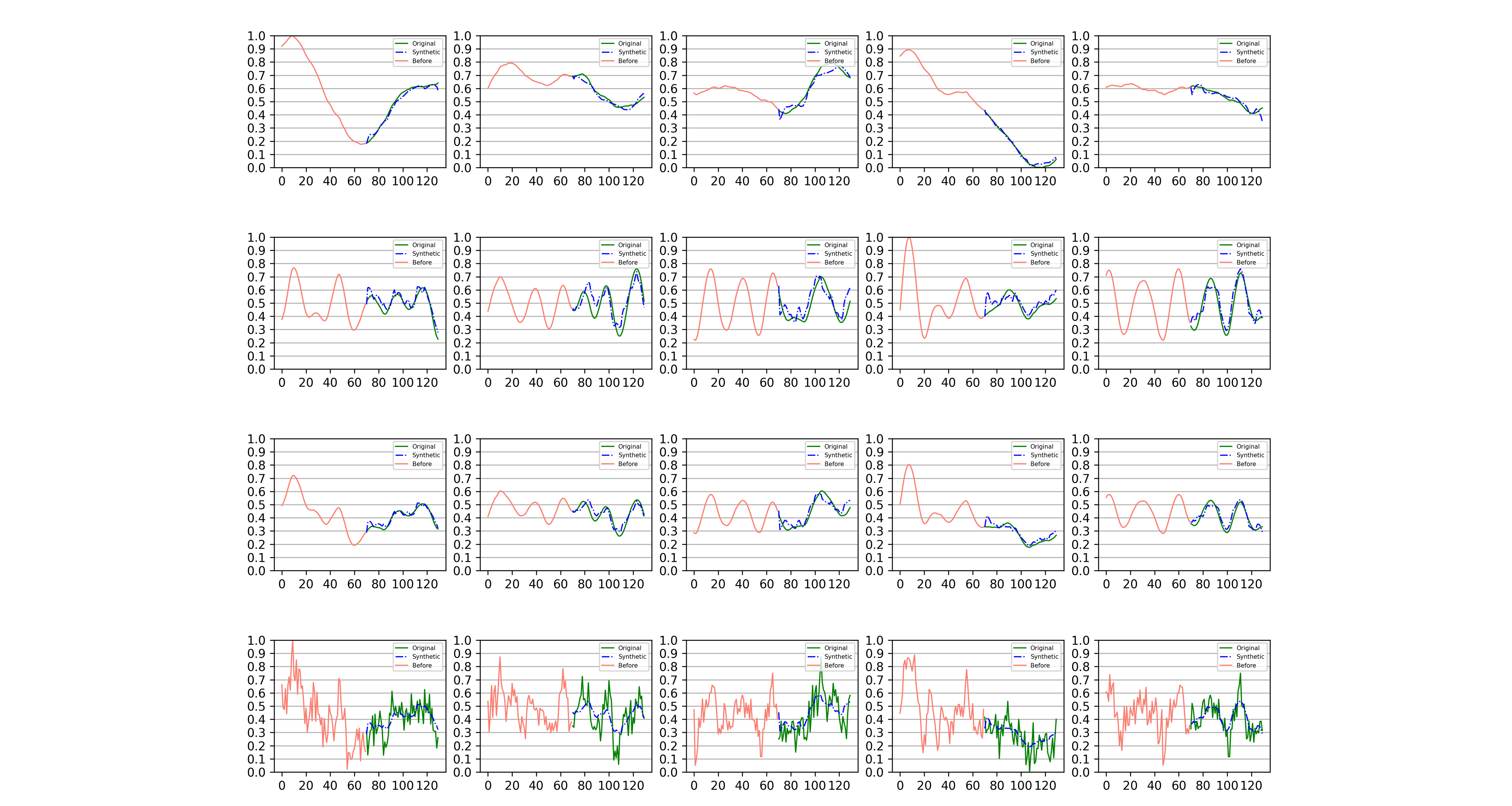
**

**Fig. S2. Comparison of real and predicted curves of component prediction results and reconstruction results.**

1. Ablation experiments on the longest predicted length


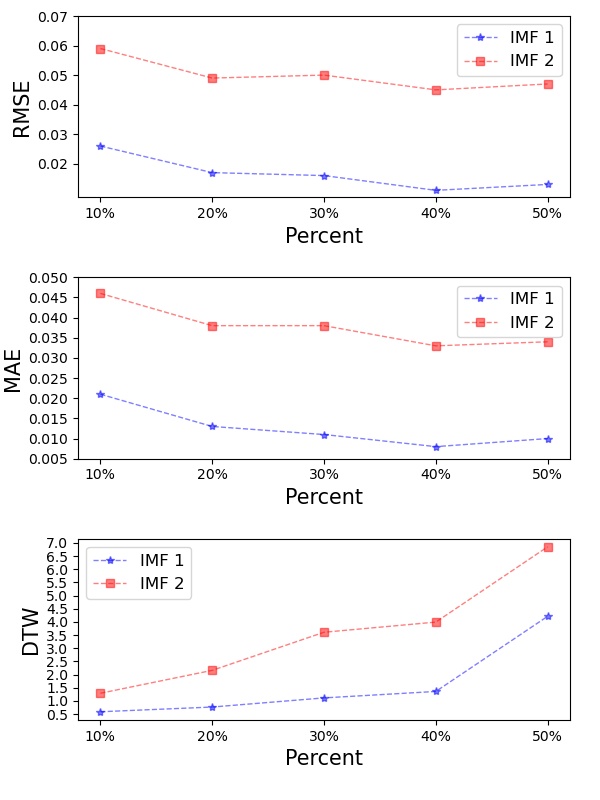


**Fig. S3. Ablation experiments on the longest predicted length.**

1. Ablation experiments on the minimum training set length


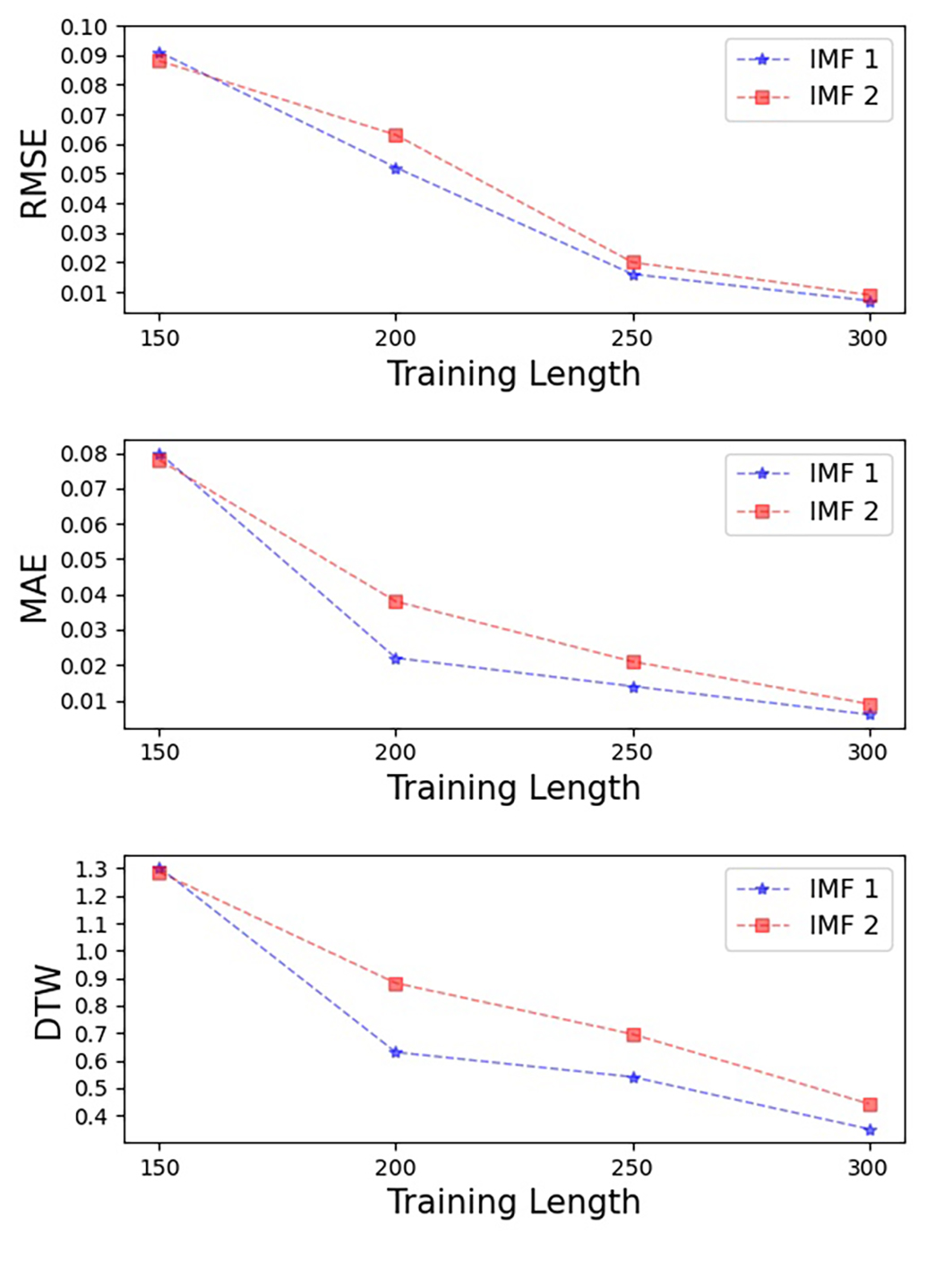


**Fig. S4. Ablation experiments on the minimum training set length.**

1. Experiments of the number of IMFs

By combining IMF 1 and IMF 2 of the original sequence as the reconstruction results and comparing them with the original series, we show the effect of different K values on reconstruction completeness. Specifically, we visualize the reconstruction results of a random subject and the original series in a picture and calculate the reconstruction errors based on RMSE and MAE and the ratio of the power spectrum of the reconstruction results to the original series. From Fig. S1, it can be seen that the low-frequency signals with K=4-6 have the best reproduction effect and are most similar to the original series. From Table S1, K=4 is the best parameter.

1. Ablation experiments on the longest predicted length

Given the extensive number of time points in the Human Connectome Project (HCP) dataset, this experiment delved into the predictive boundaries of our model using this dataset. Fig. S3 delineates the alterations in various metrics across different prediction lengths. The horizontal axis represents the proportion of the predicted length relative to the original time series length. From the graph, it is evident that with increasing prediction length, the error consistently escalates, particularly for the Dynamic Time Warping (DTW) index. Notably, there is a significant uptick in DTW error when transitioning from 40% to 50%. Consequently, this article designates the prediction length as 40%.

1. Ablation experiments on the minimum training set length

To investigate the clinical applicability of this model, we conducted ablation experiments to determine the minimum sequence length for the training set. We maintained consistent experimental settings, including subjects, brain regions, and the number of windows. As shown in Fig. S4, the prediction error remained acceptable when the sequence length was optimized above 250 time points. We believe this is due to the longer historical time series allowing the model to capture evolving patterns more effectively, thus yielding more accurate predictions. Therefore, we proceeded with classification experiments using the site with fMRI time points ≥ 250 in the ABIDE and REST-meta-MDD databases (300 and 250 time points, respectively, for ASD and MDD).
